# A new genus and species of pycnodontid fish *Flagellipinna rhomboides*, gen. et sp. nov. (Neopterygii, Pycnodontiformes), from the Upper Cretaceous (Cenomanian) of Lebanon, with notes on juvenile form and ecology

**DOI:** 10.1080/02724634.2019.1614012

**Published:** 2019-06-10

**Authors:** John Joseph Cawley, Jürgen Kriwet

**Affiliations:** Department of Paleontology, University of Vienna, Geozentrum, Althanstrasse 14, Vienna, Austria, john.cawley@univie.ac.at; juergen.kriwet@univie.ac.at

## Abstract

The Upper Cretaceous (Cenomanian) limestone quarry of Haqel, Lebanon, is home to one of the largest diversities of fossil actinopterygians in the Mesozoic, particularly of pycnodontiform fishes. Here, we describe a pycnodontiform fish, *Flagellipinna rhomboides*, gen. et sp. nov., from this locality based on four specimens. It is considered a member of the derived family Pycnodontidae due to the presence of a postparietal process. This taxon is distinct from other pycnodontids due to its diamond-shaped body, whip-like dorsal fin, postcloacal scales with forward-pointing spines, and acute anterior profile with a concave slope, giving it a ‘hunchback’ appearance. The prognathous snout armed with molariform teeth suggests that this pycnodont preyed on a variety of shelled animals from crevices. The smallest specimen is distinct in that it has a larger orbit size, no spines on the contour scales, poorly ossified skull roof bones, a notochord partially covered by arcocentra, and lacks whip-like filament on the dorsal fin, which suggest that it is a juvenile/subadult. The differences between the juvenile/subadult and other larger specimens suggest a change in ecological niche occupation during ontogeny, going from a generalized forager that lived in complex, reef habitats to moving into deeper waters to feed from crevices on the reef edge. These findings provide a more complete picture of the possible life history strategies that pycnodontiforms may have used in order to exploit different resources throughout their lives.

## INTRODUCTION

Pycnodontiformes, or pycnodonts, were a successful order of neopterygian fishes with a rich fossil record that ranged from the Late Triassic to the Eocene, ca. 175 million years (Tintori, 1981; Poyato-Ariza and Wenz, 2002; Kriwet and Schmitz, 2005). Most members are laterally compressed, with a mostly durophagous mode of feeding, and were predominant in marine habitats but also occupied estuarine and even freshwater environments (Longbottom, 1984; Poyato-Ariza et al., 1998). Although pycnodonts experienced a steady increase in diversity throughout the Mesozoic, it was the Cenomanian of Lebanon where they seemingly reached their peak in both diversity and disparity (Taverne and Capasso, 2013a, 2014a, 2015a; Marramà et al., 2016), with three families being endemic to this time and place in geological history: Coccodontidae (Taverne and Capasso, 2014b), Gebrayelichthyidae (Nursall and Capasso, 2004; Taverne and Capasso, 2014c), and Gladiopycnodontidae (Taverne and Capasso, 2013a, 2014a, 2015a). This peak in diversity may have been a result of sea level rise (Haq et al., 1987; Cavin et al., 2007) and increased sea temperature (Gale, 2000), which would have been triggered by oceanic crust production (Seton et al., 2009). These environmental changes were common events during the Cenomanian, leading to new adaptive opportunities for pycnodont speciation. Haqel is one of the most productive of these Late Cretaceous fossil sites in Lebanon (Hückel, 1970; Capasso et al., 2010; Gayet et al., 2012), with new species of fossil fishes being discovered almost every year, particularly pycnodonts (Poyato-Ariza and Wenz, 2005; Capasso et al., 2009, 2010; Taverne and Capasso, 2015b).

In this paper, we present and discuss a new genus and species of pycnodont fish, *Flagellipinna rhomboides*, gen. et sp. nov., based on a part and counterpart specimen (MNHN.F.HAK1972a and MNHN.F.HAK1972b) that was collected by the late Prof. Camille Arambourg in 1961 from the upper Cenomanian limestones of Haqel. Two other specimens of this taxon were donated by a private collector to the Muséum national d’Histoire naturelle, Paris in 2002. A similar specimen from Haqel to the ones we are studying in this paper is figured on page 93 of Gayet et al. (2012). The high quality of preservation in one particular specimen (MNHN.F.HAK2003) allows for the opportunity to discuss where it fits systematically within the Pycnodontiformes. Another of these specimens (MNHN.F.HAK2001) has characteristics that hint at this specimen being a late juvenile/subadult. The possible morphological and ecological changes undergone by this taxon during ontogeny are also discussed in this paper.

**Institutional Abbreviations**—**IPFUB**, Institut fur Paläontologie, Freie Universitat, Berlin, Germany; **MNHN**, Muséum national d’Histoire naturelle, Paris, France; **NSM**, National Museum of Nature and Science, Tokyo, Japan.

## MATERIALS AND METHODS

**FIGURE 1.**
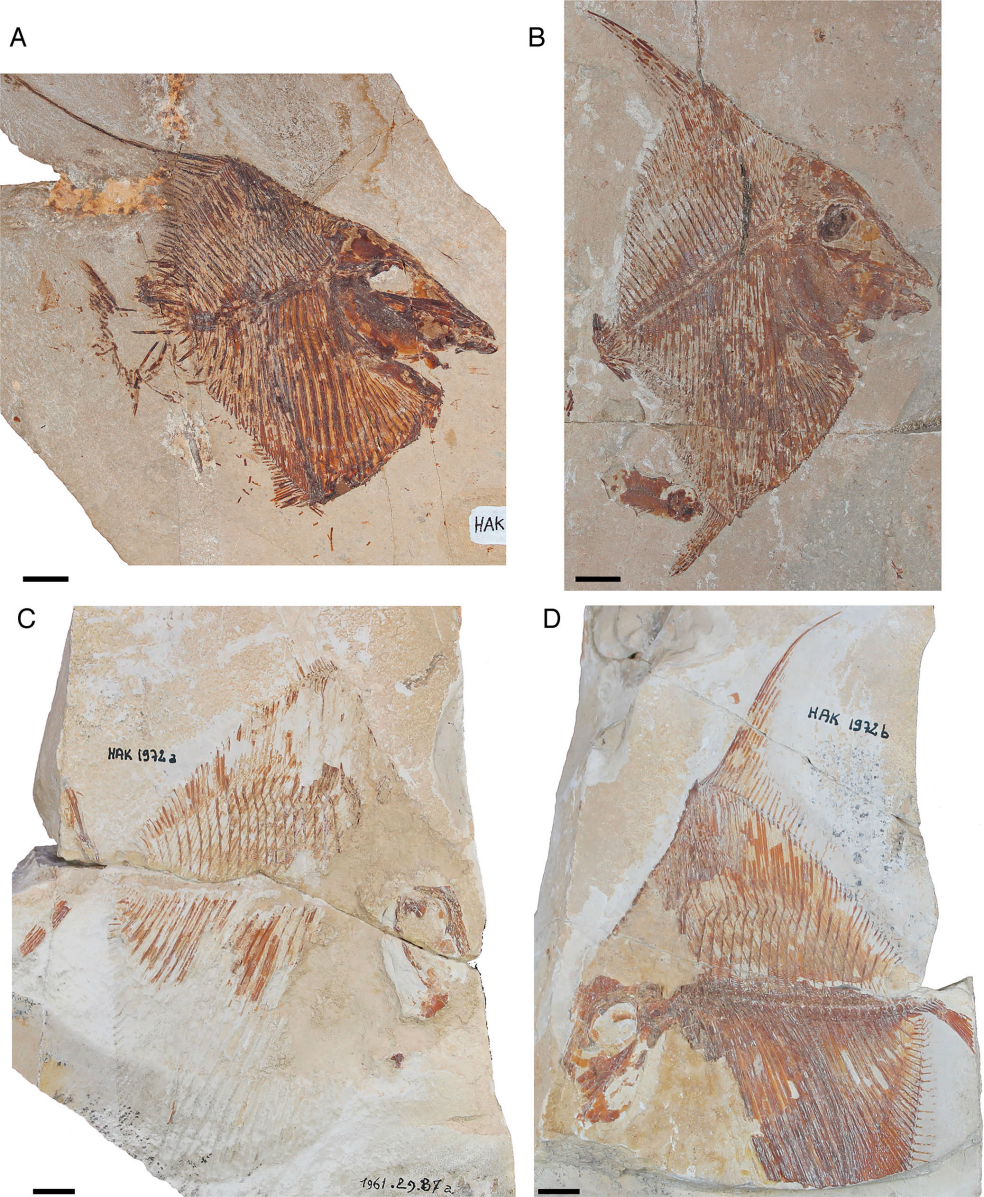
Specimens of *Flagellipinna rhomboides*, gen. et sp. nov. **A**, MNHN.F.HAK2003, holotype. **B**, MNHN.F.HAK2001, paratype, possible late juvenile/subadult stage. **C**, MNHN.F.HAK1972a, paratype, part. **D**, MNHN.F.HAK1972b, paratype, counterpart. Scale bars equal 1 cm.

*Flagellipinna rhomboides*, gen. et sp. nov., is represented by four specimens (Fig. 1) housed in the fossil fish collection of the Muséum national d’Histoire Naturelle Paris, France (MNHN.F.HAK). All specimens are from Haqel in northern Lebanon. For detailed information about the geological and stratigraphic context, see Patterson (1967), Hückel (1970), and Forey et al. (2003).

The best-preserved specimen, MNHN.F.HAK2003 (Fig. 1A), has been made the holotype because most of its anatomy, particularly that of the skull, is more or less complete. Whereas MNHN.F.HAK2001 (Fig. 1B) also has generally good preservation, its morphological characters suggest that it is not a mature specimen and represents a juvenile/subadult stage of this taxon. MNHN.F.HAK1972a and MNHN.F.HAK1972b (Fig. 1C, D) are part and counterpart of an individual specimen, in which the counterpart is the better preserved of the two, with the only relatively intact caudal endoskeleton in the hypodigm. Most of the skull and pectoral girdle of MNHN.F.HAK1972a and MNHN.F.HAK1972b (Fig. 1C, D) is eroded away, so no further preparation work can be done to reveal more of the anatomy. Most of the description of this taxon will be based on the holotype. All other specimens that contain additional morphological data will be explicitly mentioned in the text.

### Terminology

The traditional terminology for describing dermal skull bones of actinopterygians was based on nomenclature used to describe that of mammals and did not adhere to a framework based on homology. Establishing homology in the dermal elements in the skull of actinopterygians in view of their wide variability can lead to confusion, such as the use of different names for the same bone (see Schultze and Arsenault, 1985). In this paper, the terminology for the dermal head skeleton follows that of Jollie (1962) and Schultze (1993); the caudal skeleton follows Arratia and Schultze (1992) along with Schultze and Arratia (1986); the squamation follows Poyato-Ariza and Wenz (2002, 2005). Throughout this description, the traditional terms will be mentioned in parentheses alongside the current anatomical term when it is mentioned the first time.

The term ‘pycnodont’ refers to any member of Pycnodontiformes, and the term ‘pycnodontid’ refers to members of the family Pycnodontidae.

## SYSTEMATIC PALEONTOLOGY

Class OSTEICHTHYES Huxley, 1880

Subclass ACTINOPTERYGII Cope, 1887

Series NEOPTERYGII Regan, 1923

Order PYCNODONTIFORMES Berg, 1937

Family PYCNODONTIDAE sensu Nursall, 1996

*FLAGELLIPINNA*, gen. nov.

(Figs. 1–9)

**Diagnosis**—As for type and only species.

**FIGURE 2.**
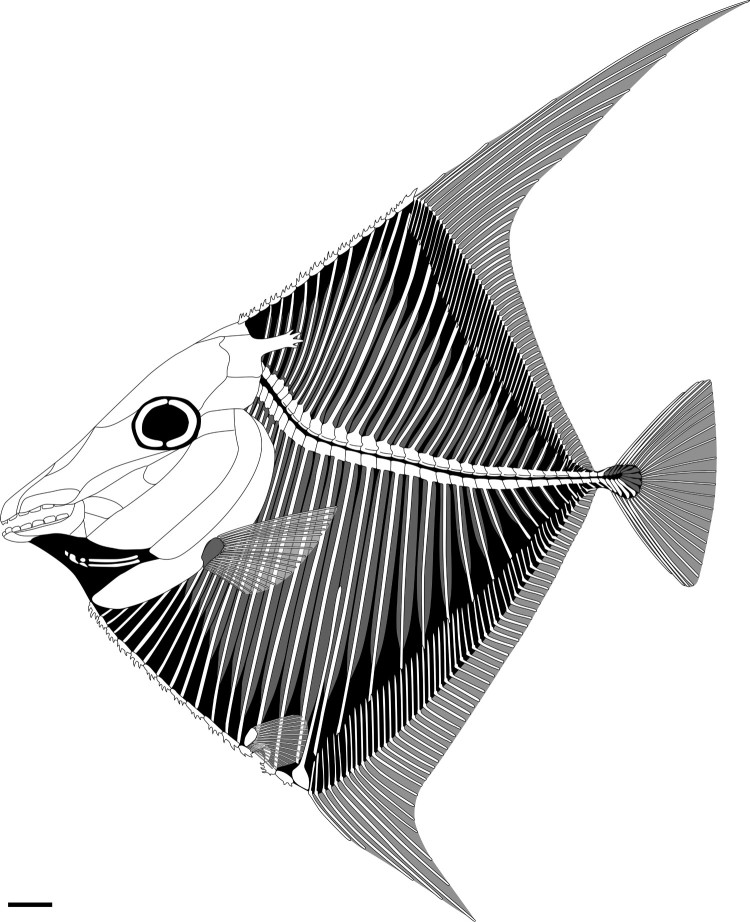
*Flagellipinna rhomboides*, gen. et sp. nov., reconstruction of the skeleton based on MNHN.F.HAK1972a, MNHN.F.HAK1972b, MNHN.F.HAK2001, and MNHN.F.HAK2003. Anatomical characters based on the holotype and the paratypes; skull roof restored from the holotype, whereas the lower jaw is based on both the holotype and MNHN.F.HAK2001; pectoral girdle is intermediate between the conditions seen in the holotype and MNHN.F.HAK2001; dorsal ridge scale series based mainly on MNHN.F.HAK1972b; caudal endoskeleton based on a combination of MNHN.F.HAK1972b and specimen figured on page 93 of Gayet et al. (2012); arcocenta based on paratype MNHN.F.HAK1972b. Reconstruction of paired fins and the caudal fin is hypothetical because they are poorly preserved in all specimens. Caudal endoskeleton that is not preserved in any specimen is represented by hypothethical bones shown here in gray. Scale bar equals 1 cm.

**FIGURE 3.**
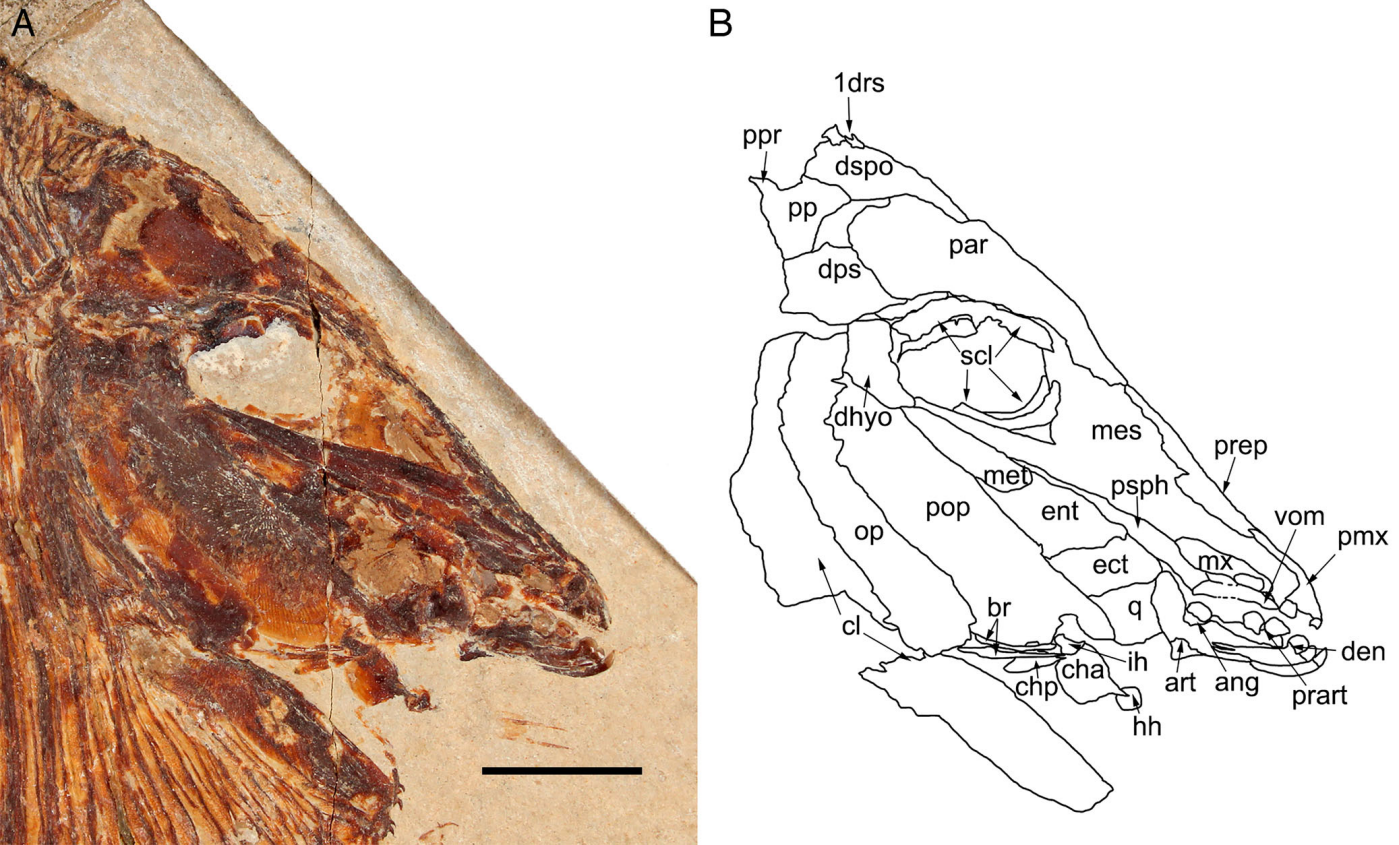
*Flagellipinna rhomboides*, gen. et sp. nov., MNHN.F.HAK2003, holotype, skull and pectoral girdle. **A**, photograph of the head and anterior region of the body. Photo by J.J.C. **B**, camera lucida drawing of the skull bones seen in **A**. **Abbreviations**: **1drs**, first dorsal ridge scale; **ang**, angular; **art**, articular; **br**, branchiostegal rays; **cha**, anterior ceratohyal; **chp**, posterior ceratohyal; **cl**, cleithrum; **den**, dentalosplenial; **dhyo**, dermohyomandibular; **dps**, dermopterosphenotic; **dspo**, dermosupraoccipital; **ect**, ectopterygoid; **ent**, entopterygoid; **hh**, hypohyal; **ih**, interhyal; **mes**, mesethmoid; **met**, metapterygoid; **mx**, maxilla; **op**, operculum; **par**, parietal; **pmx**, premaxilla; **pop**, preoperculum; **pp**, postparietal; **ppr**, postparietal process; **prart**, prearticular; **prep**, preparietal; **psph**, parasphenoid; **q**, quadrate; **scl**, sclerotic ring; **vom**, vomer. Scale bar equals 1 cm.

**FIGURE 4.**
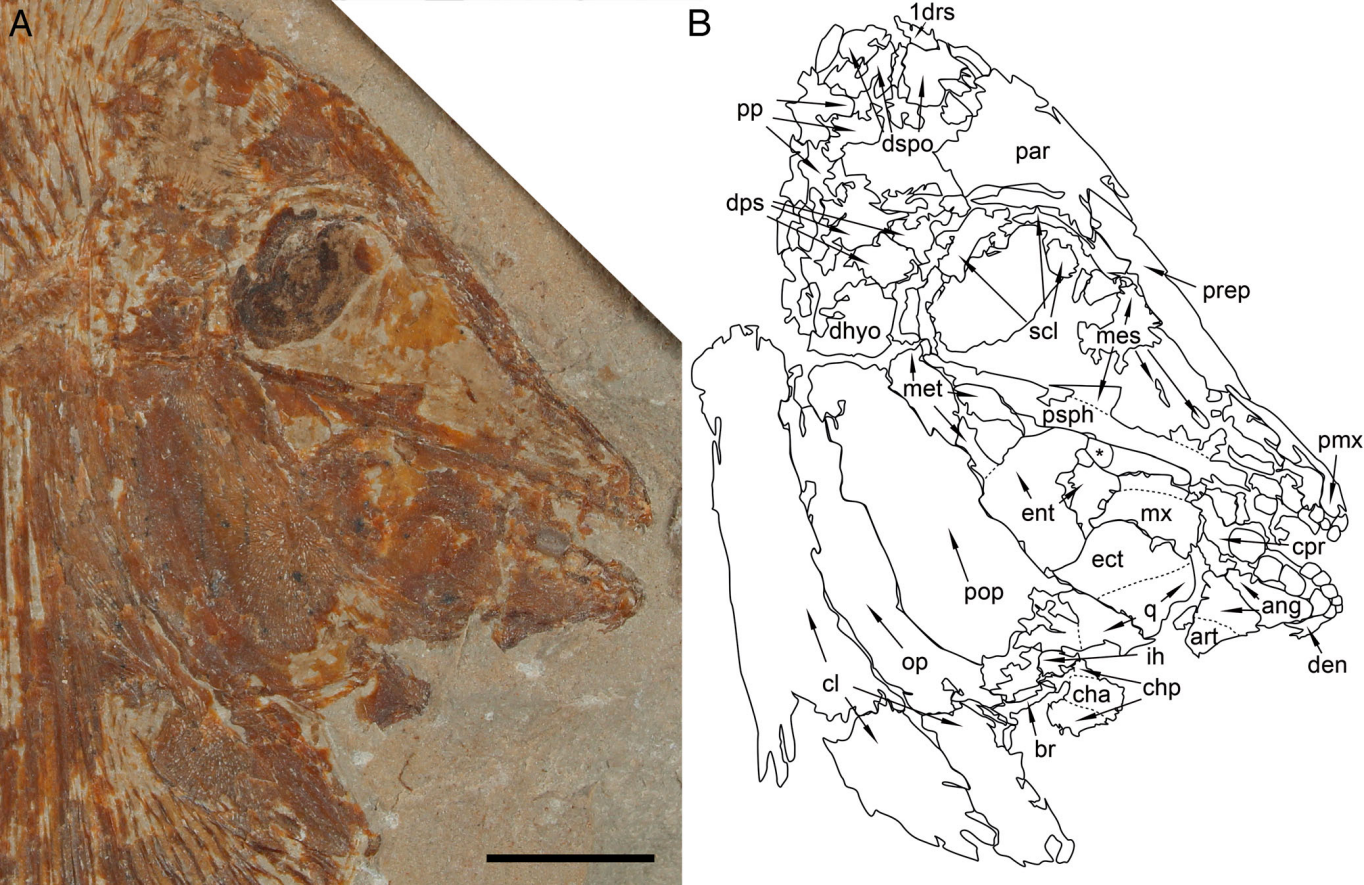
*Flagellipinna rhomboides*, gen. et sp. nov., MNHN.F.HAK2001, skull and pectoral girdle of juvenile/subadult specimen. Dotted lines indicate where sutures between bones could be visible but are difficult to confirm fully in the specimen. Ossification is poor in the skull roof, so arrows are used frequently to show what parts of fragmented material could belong to certain bones. Asterisk used to show displaced vomerine tooth overlapping ventral margin of parasphenoid. **A**, photograph of the head and anterior region of the body. Photo by J.J.C. **B**, camera lucida drawing of the skull bones seen in **A**. **Abbreviations**: **1drs**, first dorsal ridge scale; **ang**, angular; **art**, articular; **br**, branchiostegal rays; **cha**, anterior ceratohyal; **chp**, posterior ceratohyal; **cl**, cleithrum; **cpr**, coronoid process; **den**, dentalosplenial; **dhyo**, dermohyomandibular; **dps**, dermopterosphenotic; **dspo**, dermosupraoccipital; **ect**, ectopterygoid; **ent**, entopterygoid; **ih**, interhyal; **mes**, mesethmoid; **met**, metapterygoid; **mx**, maxilla; **op**, operculum; **par**, parietal; **pmx**, premaxilla; **pop**, preoperculum; **pp**, postparietal; **prep**, preparietal; **psph**, parasphenoid; **q**, quadrate; **scl**, sclerotic ring. Scale bar equals 1 cm.

**FIGURE 5.**
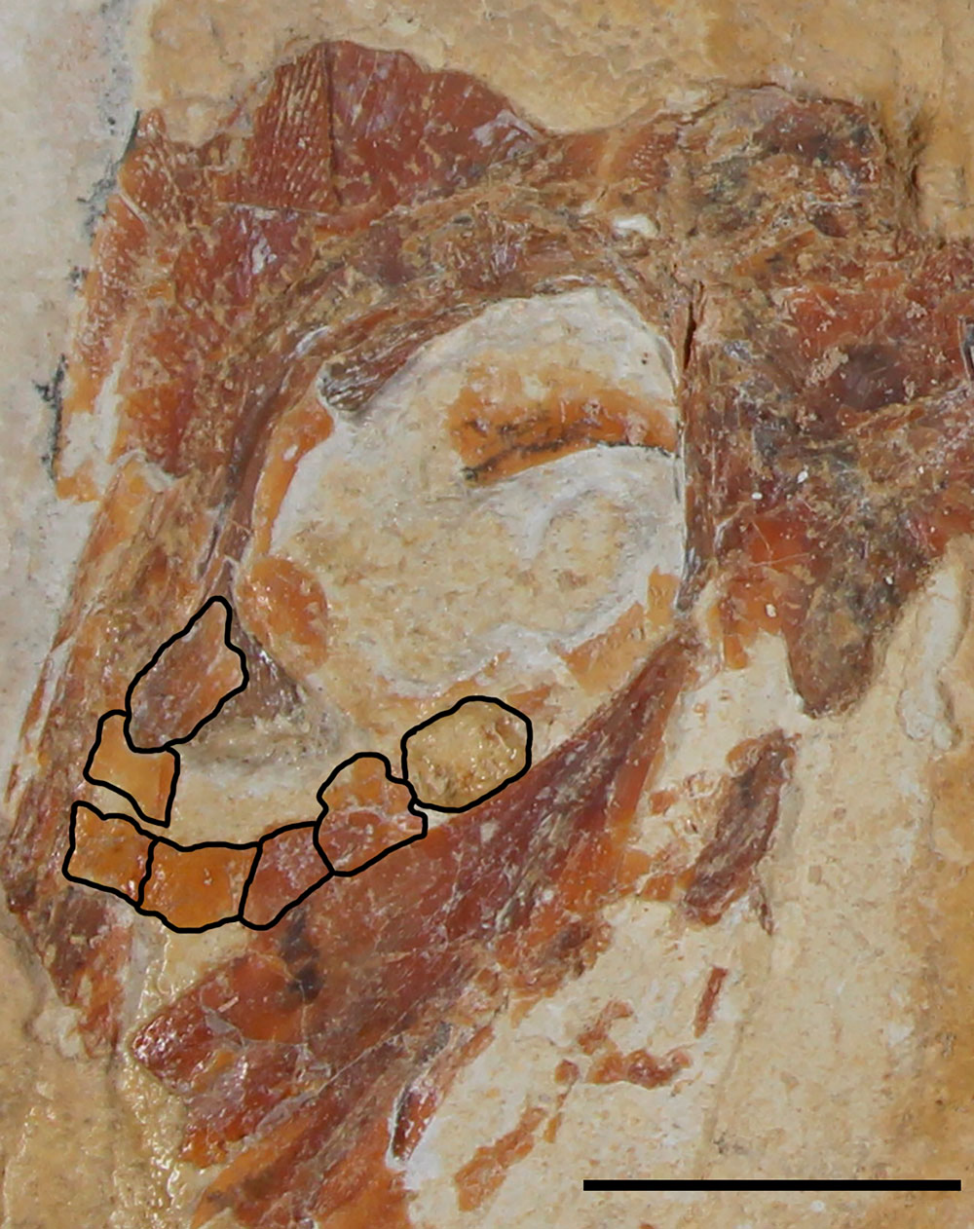
*Flagellipinna rhomboides*, gen. et sp. nov., MNHN.F.HAK1972b, paratype, infraorbital series. Scale bar equals 1 cm.

**FIGURE 6.**
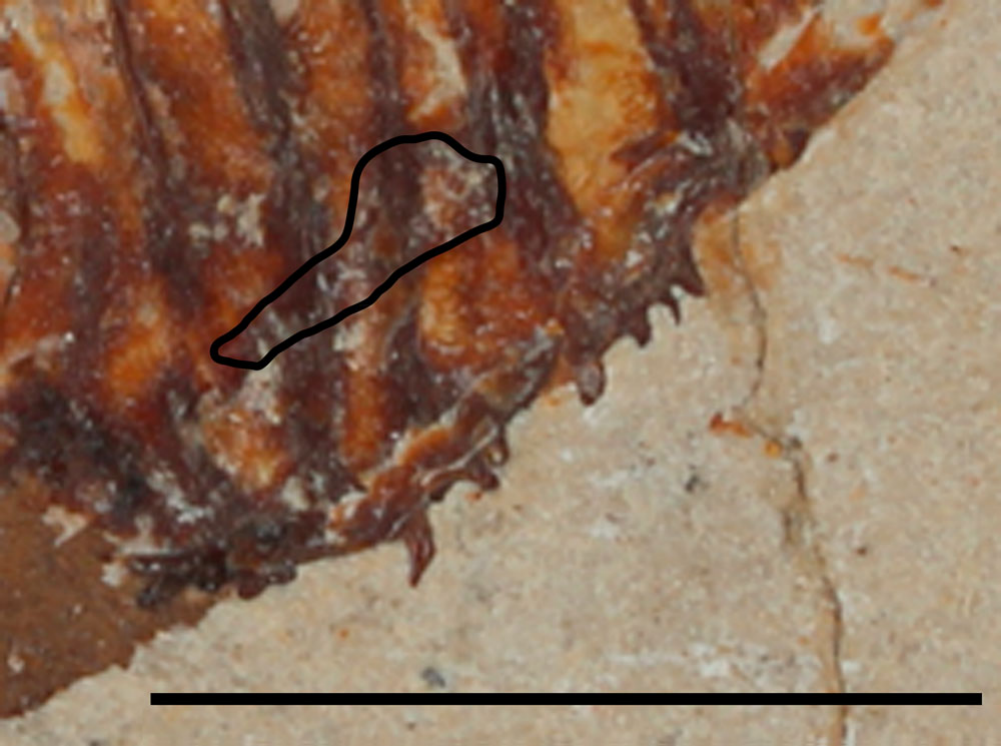
*Flagellipinna rhomboides*, gen. et sp. nov., MNHN.F.HAK2003, holotype, remains of pelvic girdle. Scale bar equals 1 cm.

**FIGURE 7.**
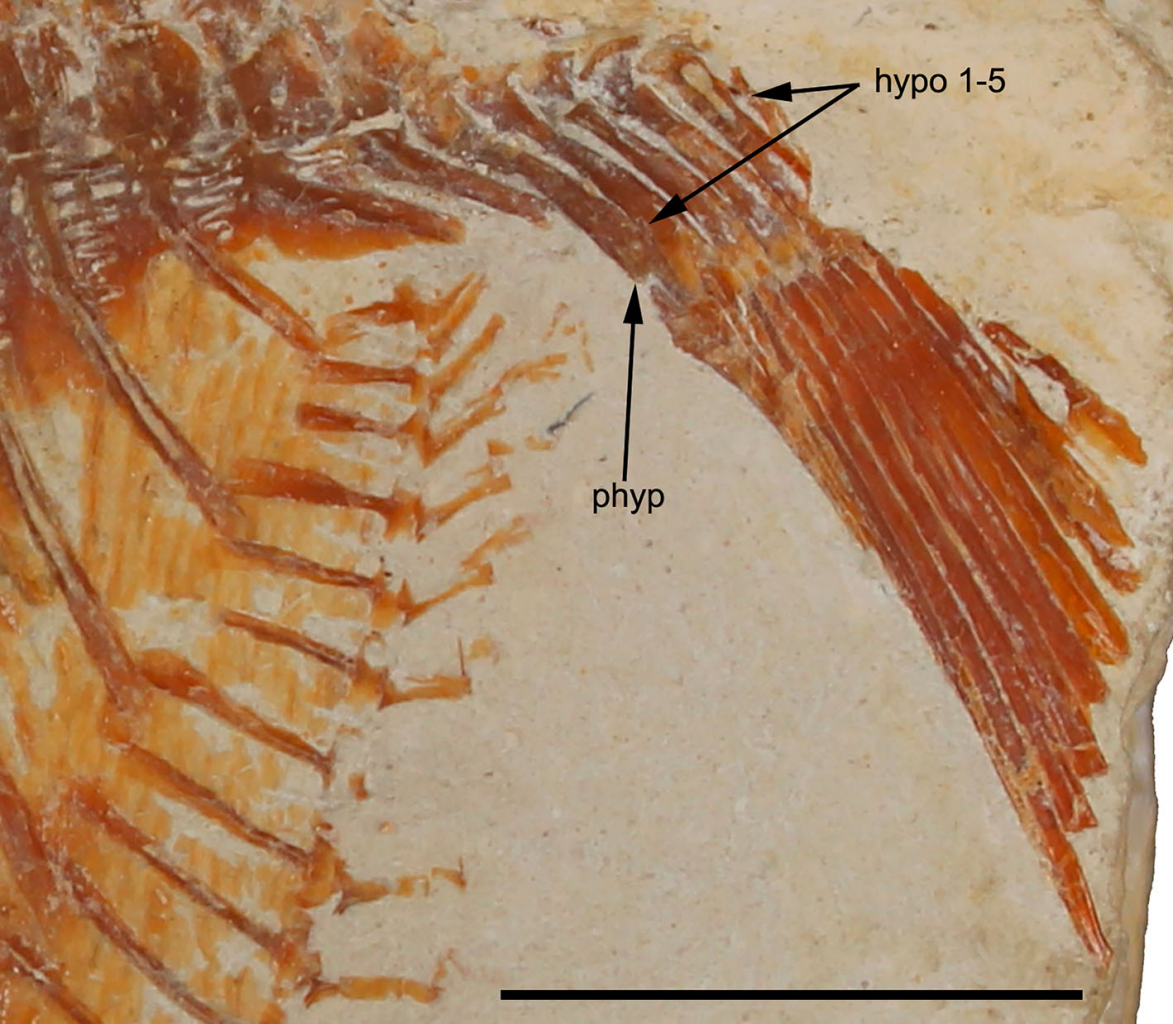
*Flagellipinna rhomboides*, gen. et sp. nov., MNHN.F.HAK1972b, paratype, caudal endoskeleton. **Abbreviations**: **hypo 1–5**, hypochordals 1–5; **phyp**, parhypural. Scale bar equals 1 cm.

**FIGURE 8.**
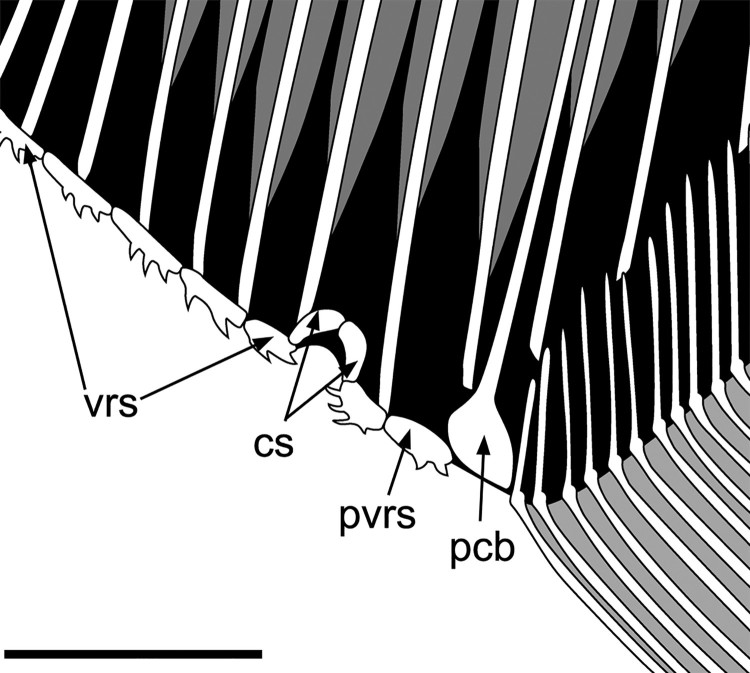
*Flagellipinna rhomboides*, gen. et sp. nov. Reconstruction of the ventral ridge scales and cloaca based on the holotype MNHN.F.HAK2003. **Abbreviations**: **cs**, cloacal scales; **pcb**, post coelomic bone; **pvrs**, postcloacal ventral ridge scale; **vrs**, ventral ridge scales. Scale bar equals 1 cm.

**FIGURE 9.**
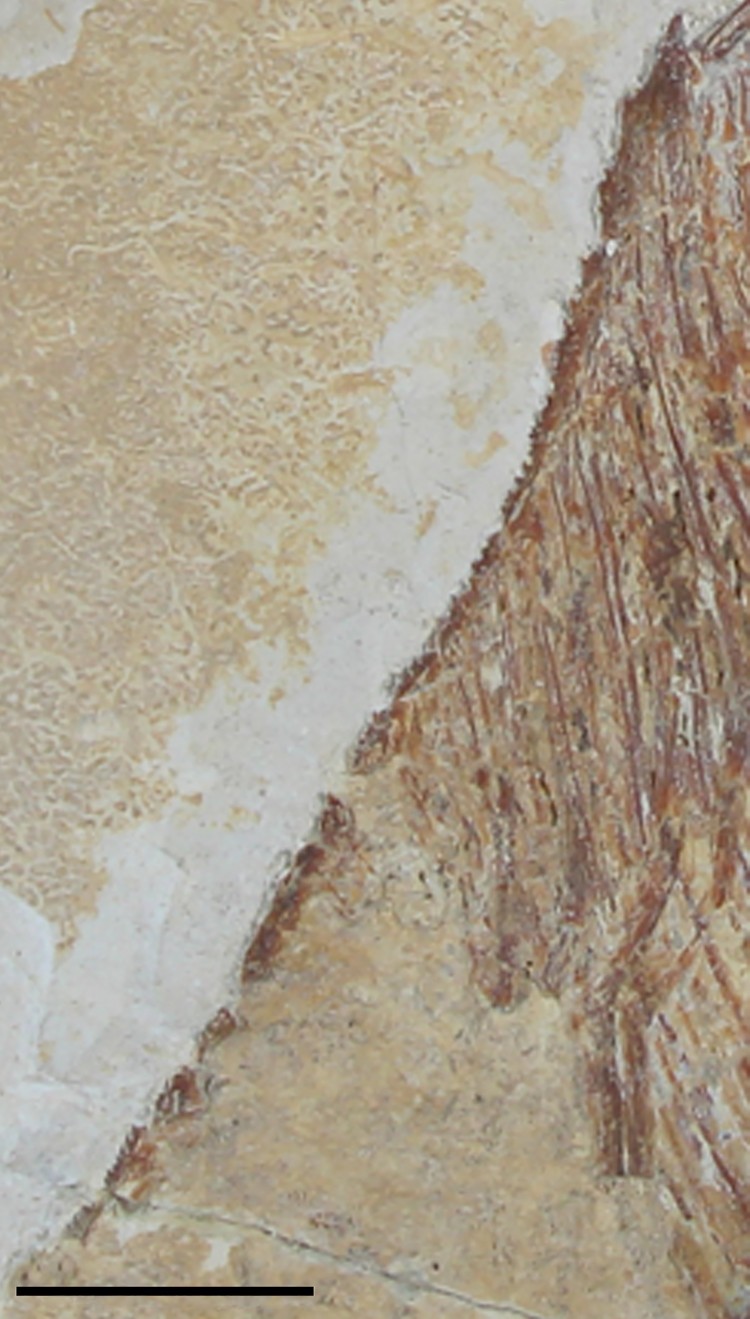
*Flagellipinna rhomboides*, gen. et sp. nov., MNHN.F.HAK1972b, paratype, dorsal ridge scales. Scale bar equals 1 cm.

**Age**—Early late Cenomanian, Late Cretaceous.

**Type Species**—*Flagellipinna rhomboides*, sp. nov.

**Etymology**—The name is a combination of the Latin words ‘flagellum’ (whip) and ‘pinna’ (fin), which refers to the whip-like dorsal fin present in this genus.

*FLAGELLIPINNA RHOMBOIDES*, sp. nov.

**Diagnosis**—Small pycnodontid fish with a deep, rhomboid body shape. Anterior profile of the fish is extremely steep, being sloped at a mean 57.3° angle in relation to the vertebral column. Dorsal fin has a whip-like filament. Dorsal and anal pterygiophores inserted very deeply into the body. Dorsal and anal fins falcate anteriorly and become progressively strap-like posteriorly. Ventral apex present anterior to the insertion of anal fin. Two dentalosplenial (dentary) teeth are broad and incisiform, and the anterior (mesial) premaxillary tooth is bicuspid. Skull dorsoventrally flattened and obliquely oriented, with an elongate snout. Paired preparietal (prefrontal) bone present. Dermocranial fenestra absent in skull roof. Cleithrum narrow and elongate with just two limbs, the dorsal limb being far narrower than the ventral limb. Large, semicircular preoperculum with small exposed dermohyomandibular. Comparatively large operculum broad and club-shaped. Notochord completely surrounded by arcocentra. Ten to 15 dorsal ridge scales with three to six backward-pointing spines. Ventral ridge scales consist of 11 precloacal scales, with two backward curved spines and a single postcloacal scale. Cloaca is roofed by two comma-shaped scales. Four spines present on the postcloacal ventral ridge scale, with the first three being strongly curved and forward pointing and the most posterior spine straight and backward pointing in a posteroventral angle. Complete scales restricted to abdominal region, scale bars on rest of body.

**Holotype**—MNHN.F.HAK2003, a nearly complete skeleton (Fig. 1A).

**Paratypes**—MNHN.F.HAK2001, the skeleton of a possible late juvenile/subadult fish; MNHN.F.HAK1972a and MNHN.F.HAK1972b, part and counterpart of a nearly complete fish.

**Type Locality and Horizon**—Haqel, northern Lebanon; Sannine Formation, early late Cenomanian, Upper Cretaceous.

**Etymology**—Latin for ‘rhomboid’ in allusion to the diamond-like body shape of the new species.

## DESCRIPTION

### General Morphology, Size, and Ontogenetic Stage

*Flagellipinna rhomboides*, gen. et sp. nov., is a small pycnodont, with the largest, MNHN.F.HAK1972, having a standard length (SL) of 9.72 cm and the smallest, MNHN.F.HAK2001, having a SL of 7.04 cm (Table 1). Due to the incomplete preservation of MNHN.F.HAK1972, its true standard length is significantly underestimated. Based on the specimens in this study, the mean SL for this taxon is 8.33 cm.

**TABLE 1.** Relevant measurements (in cm), proportions, and meristic characters of the articulated specimens of the type series of *Flagellipinna rhomboides*, gen. et sp. nov.

Dimension	Holotype	MNHN.F.HAK2001	MNHN.F.HAK1972
SL	8.23	7.04	9.72
PDOR	6.28	5.33	3.47
%SL	76.30	75.70	35.70
PANA	4.31	4.60	—
%SL	52.40	65.30	—
PPEC	2.92	2.87	—
%SL	35.50	40.80	—
HD	4.09	5.17	—
%SL	49.70	73.40	—
MBD	8.02	10.07	—
%SL	97.40	143.00	—
%HD/MBD	51.00	51.30	—
HL	4	3.54	—
%SL	48.60	50.30	—
DFH	2.05	1.12	4.39
%SL	24.90	15.90	45.20
%HD/HL	103.30	146.00	—
DP	(36)	38	46
AP	(31)	(38)	46
VE	(22)	27	(24)
AE	7	5	—
AS	6	4	—
DRS	10	(17)	(24)
VRS	12	14	—
PCL	1	2	—
ACS	1	1	—
PCS	1	—	—
°APROF	47.60	65.79	58.51

Parentheses indicate that the corresponding measure is an estimate or that the figure is based on a poorly preserved structure. All SLs are estimated measurements due to incomplete preservation in all specimens. **Abbreviations**: **°APROF**, slope of anterior profile; **%HD/HL**, percentage of ratio between head depth and head length; **%HD/MBD**, percentage of ratio between head depth and maximum body length; **%SL**, percentage of ratio between standard length and above measurement; **ACS**, number of anterior modified cloacal scales; **AE**, number of abdominal vertebrae (epaxial elements); **AP**, number of anal pterygiophores; **AS**, number of anterior autogenous neural spines; **DFH**, dorsal fin height; **DP**, number of dorsal pterygiophores; **DRS**, number of dorsal ridge scales; **HD**, head depth (from highest point of dermosupraoccipital to lowest point of cleithrum); **HL**, head length (from anterior border of premaxillary to posterior border of cleithrum); **MBD**, maximum body depth; **PANA**, preanal distance; **PCL**, number of postcloacal ventral ridge scales; **PCS**, number of posterior modified cloacal scales; **PDOR**, predorsal distance; **PPEC**, prepectoral distance; **SL**, standard length; **VE**, number of vertebrae (epaxial elements excluding those supporting precurrent and principal caudal fin rays); **VRS**, total number of ventral ridge scales.

The species has a deep, diamond-like body shape (Figs. 1, 2), which can be confirmed by the height/standard length ratio, with the lowest being 97.4% in the holotype. All relevant morphometric measurements along with meristic characters are given in Table 1. The new species also is most noticeable by its acute anterior profile (the region of the body measured between the tip of the premaxilla and the anterior dorsal fin ray) with a slightly concave slope, giving it a ‘hunchback’ appearance. The holotype has the most extreme acute anterior profile of all the specimens studied with a slope of 47.6°. The dorsal apex is just anterior to the insertion of the dorsal fin, which can be demonstrated by the large last dorsal ridge scale in MNHN.F.HAK1972b. The ventral apex is marked by a large postcloacal ventral ridge scale. The dorsal and anal fins start out falcate and become increasingly strap-like in the posterior direction. Due to the deep, diamond-shaped body, these fins have a very inclined line of insertion with the body, which converges toward a very short but distinct caudal peduncle. Paired fins and caudal fins are insufficiently preserved to provide a realistic indicator of either size or morphology, with pelvic fins entirely absent from the material studied.

The head is relatively long, constituting up to half of the standard length in MNHN.F.HAK2001. The head makes up to half of the maximum body depth, ranging from 51% to 51.3% in the three specimens (Table 1). The head is only slightly deeper than it is long, with a considerably elongate snout in the holotype, giving it a very gracile appearance in contrast to the condition in MNHN.F.HAK2001, which exhibits a skull that is significantly deeper than long (Table 1).

The differences in head depth between the holotype and MNHN.F.HAK2001, along with characteristics such as contour scales without spines and poor ossification in the skull roof, are interpreted here to be signs of MNHN.F.HAK2001 being a juvenile specimen (see Discussion). All other specimens appear to be adult based on the degree of cranial and vertebral element ossifications.

### Skull

**Endocranium**—The endocranium in pycnodontiforms is predominantly cartilaginous and normally poorly preserved. The same is true for all three specimens of the new taxon, and no endochondral structures are unambiguously identifiable.

The mesethmoid is a large, wedge-shaped ossification (Figs. 2, 3) anterior and ventral to the orbit that covers the area of snout between the parasphenoid and the preparietal. The mesethmoid anterior to the orbit is less ossified than the heavily ossified ventral portion. In MNHN.F.HAK2001, the mesethmoid is less ossified, particularly in the area anterior to the orbit and posterior to the premaxilla, another indication that this specimen could be a juvenile (Fig. 4).

**Skull Roof**—The best-preserved skull roof is seen in the holotype (Fig. 3). In the juvenile specimen (Figs. 1B, 4), MNHN.F.HAK2001, bones of the skull roof are difficult to make out but can be discerned by how the striations on the bone spread out. The first dorsal ridge scale is very close, but not fused, to the dermosupraoccipital at the posterior margin of the skull roof in the holotype, whereas it is more clearly separated from the posterior skull margin in MNHN.F.HAK2001. It is a small, triangular bone with two backward-pointing spines located on the dorsal margin of the scale. It overlaps the dermosupraoccipital, a triangular bone that occupies the posterodorsal corner of the skull roof. The dermosupraoccipital in MNHN.F.HAK2001 has a rectangular shape with a concave anterior margin.

The postparietal (parietal) is located ventral to the dermosupraoccipital and posterior to the parietal (frontal). It is a broad, rectangular bone with a large, triangular anterodorsal extension and a slightly concave notch located ventral to the extension to accommodate the parietal. Posterior to the postparietal is the postparietal process (parietal peninculus of Nursall, 1996). The number of posterior extensions in the process is hard to identify because they either appear to be fused together or are imperfectly preserved. Its shape is similar to that of the dermosupraoccipital in MNHN.F.HAK2001. A small extension appears from the posterior margin of the postparietal, and this is indicative of the postparietal process, but it is very faintly preserved.

A trapezoidal bone ventral to the postparietal and posterior to the parietal is the dermopterosphenotic. In MNHN.F.HAK2001, the dermopterosphenotic is more problematic to discern but is similarly broad, as can be seen in the holotype (Fig. 4). The parietal covers the area dorsal and anterior to the orbit. It is a narrow bone anterior to the orbit that broadens significantly posteriad.

The preparietal is a triangular bone that lies ventral to the parietal and is overlain by the premaxilla dorsal process anteriorly due to taphonomic processes. It is an elongate bone that narrows to a point as it touches the premaxillary process in MNHN.F.HAK2001.

The orbit is large (diameter 86 mm), contributing 10.45% of SL, and a great distance between the tip of the premaxilla and the orbit (1.65 cm or 20.05% of SL) gives the snout its elongate shape. The crescent-shaped ossifications present ventral to the parietal and anterior to the dermopterosphenotic are the sclerotic bones that make up the orbit. MNHN.F.HAK2001 has the largest orbit (orbit diameter being 97 mm), with long, curved sclerotic bones present in the dorsal region of the orbit, making up to 13.78% of SL. Additional sclerotic bone elements are present in the anterodorsal region of the orbit in MNHN.F.HAK1972b. A large orbit size is a feature usually associated with juvenile actinopterygians and also has been demonstrated for another pycnodontid fish, *Pycnodus* (Cawley et al., 2018). In contrast to the holotype, the preorbital distance is shorter at 12.2 mm (17.21% of SL), giving the head profile a steeper appearance.

The infraorbital series is preserved in MNHN.F.HAK1972b (Fig. 5) in the form of six bones, with the infraorbitals most anteroventral to the orbit having a thickened posterior margin. This series of bones forms a ‘V’-shaped formation anteroventral to the orbit. These infraorbitals are distorted by taphonomic processes, however, so their true shape is hard to discern, but the posterior-most infraorbital has a rectangular form.

The dermal skull of MNHN.F.HAK1972b is poorly preserved, but what remains is similar to that seen in the holotype.

**Parasphenoid**—The parasphenoid is an elongate bone with a strongly backward-curving dorsal process lying parallel to the mesethmoid and posterior to the vomer (Fig. 3). As the parasphenoid gets closer to the posteroventral margins of the orbit, it becomes completely overlapped by the mesethmoid. Unlike in the holotype, the parasphenoid in MNHN.F.HAK2001 is at a more obtuse angle, becoming broader posteriad (Fig. 4).

**Jaws and Vomer**—The jaw apparatus of the new taxon corresponds to the general pycnodont pattern. The toothless maxilla is preserved as an elongate bone with an expanded posterior extension and a concave ventral notch, giving it an elongate reniform shape in the new genus. In MNHN.F.HAK2001, the maxilla, which also is elongate, having convex margins, is displaced posterior to the coronoid process (Fig. 4).

The premaxilla bears two labiolingually flattened and mesiodistally elongate incisiform teeth with differing morphologies. The anterior (mesial) tooth is bicuspid, whereas the larger posterior (lateral) tooth is incisiform with a continuous apical cutting edge. The premaxilla has an elongate, dorsally directed process (processus anterior) that lies against the mesethmoid and the base of the preparietal. The anterior bicuspid tooth and a small remnant of a second tooth are preserved on the premaxillary in MNHN.F.HAK2001 (Fig. 4). The anterior tooth is incisiform, with a deeply concave apical cutting edge forming a bicuspid tooth crown.

The dentalosplenial bears two flattened incisiform teeth. The posterior portion of the dentalosplenial is deeply incised. Two incisiform teeth on the dentalosplenial have continuous, rounded apical cutting edges (Fig. 3). On the left dentalosplenial, only one tooth is preserved medially in the holotype MNHN.F.HAK2003. The posterior tooth on the right dentalosplenial is larger than the anterior tooth. The dentalosplenial has two flattened incisiform teeth, and the posterior extension of the bone is bifurcated in MNHN.F.HAK2001 (Fig. 4).

The prearticular dentition is exposed in lateral view in the holotype and MNHN.F.HAK2001. In the holotype, four large, rounded, molariform prearticular teeth of the lateral-most row lie on the dorsal margin of the prearticular and are visible in lateral view (Fig. 3). Only the right lateral row of teeth is preserved. In MNHN.F.HAK2001, only three lateral prearticular teeth are preserved (Fig. 4). The anterior-most tooth is the smallest and is typically molariform, whereas the succeeding tooth is a more elongate molariform structure. The most posterior tooth on this specimen is exposed in such a way that the occlusal surface is visible. It is large and oval in shape and without any ornamentation.

The coronoid process is well developed and displays the characteristic morphology found in most pycnodonts (Figs. 2, 3). Very little of the angular is preserved in the holotype. All that remains is a wedge-shaped bone located ventral to the prearticular and posterior to the dentalosplenial. In MNHN.F.HAK2001, the angular is ventral to the prearticular and what is preserved of it seems to indicate that it is triangular in shape. The remainder of the lower jaw is also best preserved in MNHN.F.HAK2001 (Fig. 4). What is left of the articular is a small, triangular bone, which is ventral to the angular and located in the posteroventral corner of the lower jaw.

The vomer in the holotype is an elongate, rectangular bone partially preserved anteroventral to the maxilla and anterior to the parasphenoid in lateral view (Fig. 3). There is one vomerine tooth present in the holotype overlapping the anterior portion of the maxilla. It is an elongate and oval molariform tooth with a visible occlusal surface. The best-preserved vomer is discernible in MNHN.F.HAK2001 and is an elongate bone with a posterodorsal process. An isolated vomerine tooth with a visible occlusal surface has become displaced and is located between the ventral margin of the parasphenoid and the anterior margin of the entopterygoid. Three other vomerine teeth seem to be present in the rocky matrix dorsal to the preserved prearticular tooth row. The most anterior tooth is small and rounded, whereas the two posterior teeth are elongate ovals. They are visible in the ventral view. The coronoid process is located high in posterodorsal corner of bone and has a flat dorsal margin and convex anterior and posterior margins. The vomer is not preserved in MNHN.F.HAK1972.

**Suspensorium**—The hyomandibular is covered by elements of the opercular apparatus in all three specimens so that its morphology cannot be identified. The dermohyomandibular is a wedge-shaped bone located posterior to the orbit and is exposed dorsally to the preoperculum (Figs. 2, 3). The surface displays a reticulated ornamentation. Dorsally, it forms the articulation with the neurocranium.

The preoperculum is very large and is located alongside the metapterygoid and entopterygoid. It is elongate with a convex posterior margin, which is overlapped by the operculum, but the posteroventral corner is exposed, showing its true shape. The operculum is a broad and elongate, club-shaped bone, which overlaps the posterior margin of the preoperculum and the posteroventral portion of the dermohyomandibular. The striations ornamenting the operculum face in a dorsoventral direction, whereas the striations on the preoperculum are directed more anteroposteriorly. The great width and height of this operculum makes it larger than usually seen in pycnodonts (Poyato-Ariza and Wenz, 2002).

Posterior to the overlap of the mesethmoid and the parasphenoid and anterior to the preoperculum is the metapterygoid, which is elongate and tear-shaped. The convex ventral margin of the metapterygoid overlaps the dorsal margin of the entopterygoid, which is wedge-shaped. Ventral to the entopterygoid and with a similar shape is the ectopterygoid. Ventral to the ectopterygoid, posterior to the coronoid process, and anterior to the anteroventral margin of the preoperculum is the quadrate, which is trapezoid in shape.

**Ventral Hyoid Arch**—The hypohyal is a small, rectangular replacement bone but is fragmented anteriorly, so its true shape is hard to discern. Positioned above the hypohyal is the anterior ceratohyal, which is a large, rectangular bone that has moved during burial from its original position because the heavily ossified ventral portion typical of pycnodont anterior ceratohyals faces anteriorly, whereas its less ossified dorsal region is posterad. What are interpreted to be the posteroventral extensions with a pointed distal tip overlap the posterodorsal margin of the hypohyal.

The posterior ceratohyal is more elongate, with a flat ventral margin and a convex dorsal margin. In MNHN.F.HAK2001, the posterior ceratohyal is displaced ventral to the preoperculum. It is trapezoid in shape. It is overlapped by what appears to be an anterior ceratohyal, which is rectangular in shape and would have moved posteriorly due to taphonomic processes (Fig. 4).

The interhyal is displaced from its usual position and is positioned dorsal to the anterior ceratohyal in the holotype, whereas it is located dorsal to the posterior ceratohyal in the supposed juvenile, MNHN.F.HAK2001. It is a trapezoidal bone with a convex ventral margin, and what remains of the dorsal margin is flat.

The two branchiostegal rays are thin and elongate, which is typical for pycnodonts. They are preserved lying alongside the ventral margin of the preoperculum. The ventral (right) branchiostegal ray overlaps the posterior ceratohyal, whereas the dorsal (left) branchiostegal ray is situated above and is partially overlapped by the posterior ceratohyal.

### Branchial Skeleton

Elements of the branchial skeleton are covered by elements of the opercular apparatus or are otherwise not preserved. Therefore, it is impossible to identify any branchial arches or whether branchial teeth are present.

### Paired Fins

**Pectoral Girdle**—The cleithrum is a large, rectangular bone with rounded edges in the new taxon. It is large and well ossified and located posteroventral to the operculum (Fig. 2). The dorsal and ventral limbs form an obtuse angle, with the dorsal limb continuing into a spine-like process for articulation with the supracleithrum, whereas the ventral limb is expanded and broadly rounded. The anterior margin of the cleithrum is relatively thick, in contrast to the less ossified posterior margin. The posteroventral margin is blade-like, with a notch dorsally to accommodate the pectoral fin. The supracleithrum and the posttemporal that form the articulation of the pectoral girdle with the cranium as well as the scapula and the coracoid are not preserved or are obscured by other skeletal elements.

**Pectoral Fins**—Remains of the pectoral fin are disarticulated but preserved in the notch at the confluence of the dorsal and ventral limbs of the cleithrum. It is possible to identify five or six pectoral fin rays. In the supposed juvenile specimen, MNHN.F.HAK2001, eight partially preserved, unsegmented, elongate fin rays are preserved. Ventral to these are two club-shaped pectoral radials, with three thicker, fan-shaped pectoral radials ventral to them. Taphonomic processes have shifted the location of these bones significantly, so the true arrangement of the pectoral radials remains unknown.

**Pelvic Girdle and Fin**—No pelvic fin rays have been preserved in all the specimens studied, but a structure is preserved anterior to the cloaca indicative of a pelvic girdle element (Fig. 6). The pelvic girdle is similar in shape to a right-angled triangle but also has a comparatively flat dorsal margin.

### Median Fins

**Dorsal Fin**—The dorsal fin is imperfectly preserved but seemingly was strongly falcate, with the anterior rays being very elongate (Figs. 1, 2). It diminishes in height in an anteroposterior direction, becoming progressively strap-like. At least 21 dorsal fin rays are present. Due to the disarticulation near the caudal fin, the true number of dorsal pterygiophores cannot be determined, but at least 35 or 36 are present in the holotype. The caudal region is better preserved in MNHN.F.HAK2001 and MNHN.F.HAK1972b, so the complete number of dorsal pterygiophores can be ascertained in these specimens, and amount to 38 and 46, respectively. These pterygiophores, which are broad and elongate, curve anteriorly just below the dorsal fin rays. This is most apparent in the first few pterygiophores. The pterygiophores are inserted very deeply into the body, with the deepest dorsal pterygiophore inserted 91.5 mm into the body of the holotype, 150 mm in MNHN.F.HAK2001, and 183 mm in MNHN.F.HAK1972b.

The extremely elongate, whip-like structure in the holotype, which is positioned at about the location of the fifth dorsal fin ray, appears to be made up of a series of seven elongate ray segments that intertwine with each other. However, it is likely that taphonomic processes, e.g., water currents, disarticulating the fin rays could have led to this fragmented appearance. This is further supported by the observation that the fin rays in both MNHN.F.HAK1972 and MNHN.F.HAK2001 are singular structures. The fourth dorsal fin ray is the longest fin ray in MNHN.F.HAK1972, which also shares the extremely elongate, whip-like fin rays. This structure is over 5 cm long and is four to five times longer than the second tallest dorsal fin ray. In MNHN.F.HAK2001, it is the sixth dorsal fin ray that is the longest, but it is not as whip-like as those found in the other specimens (Fig. 1B). The comparably shorter whip-like structure in this specimen might be related to the fact that it represents a juvenile.

**Anal Fin**—The anal fin also is very elongate and strongly falcate (Figs. 1, 2). There are 31 preserved anal pterygiophores in the holotype, but only 29 or 30 in MNHN.F.HAK1972b and even fewer (19) in MNHN.F.HAK2001. The rather small number of preserved pterygiophores in MNHN.F.HAK2001 might be related not only to taphonomic processes but also to the fact that it most likely represents a juvenile. Like the dorsal pterygiophores, the anal pterygiophores are deeply inserted into the body, with the longest pterygiophore inserted at a depth of 107 mm in both the holotype and MNHN.F.HAK2001 and insertion in MNHN.F.HAK1972b being only slightly deeper at 110 mm.

Not much of the anal fin is preserved in the holotype, but the posterior portion of the fin is the only part that is preserved in MNHN.F.HAK1972b and reveals that the anal fin starts out falcate and becomes more strap-like in an anteroposterior direction due to the decreasing size of the anal pterygiophores. Unlike the dorsal fin, the anal fin is consistent in overall shape in all specimens.

**Caudal Fin and Endoskeleton**—The caudal fin is very poorly preserved in all specimens. The end of the vertebral column bends in an upward arc due to taphonomic processes, and the caudal endoskeleton cannot be completely identified. There are 30 or 31 caudal fin rays preserved in the holotype. The fin rays are all elongate and unsegmented with no bifurcations. The preservation is little better in the supposed juvenile, MNHN.F.HAK2001, where what remains of the caudal fin rays is facing inward, obscuring the epichordals and hypochordals. A total of 25 caudal fin rays are preserved in this specimen, and the fin ray morphology is identical to that seen in the holotype.

It is in MNHN.F.HAK1972b where the caudal endoskeleton is preserved to some degree (Fig. 7). The parhypural is expanded at both proximal and distal ends and contracts medially. The anterior margin of the proximal tip is convex, whereas the posterior margin is concave. Where the medial section contracts, there are a slightly concave margin and a convex margin on the anterior margin and the posterior margin closest to the distal tip, respectively. Five hypochordals are present. The first two hypochordals have an expanded head, which narrows in the middle section and then broadens out to a fan. The third and fourth hypochordals are also fan-shaped at the distal end, but their median section is much broader and does not contract. Hypochordal 5 is not fully preserved, but what remains suggests its morphology to be similar to that of the first two hypochordals. No epichordals are preserved. There are 13 caudal fin rays preserved in MNHN.F.HAK1972b. These are all in the ventral lobe of the caudal fin. The tips of the fin rays are not preserved, so it remains ambiguous whether any bifurcation pattern is present or not. In MNHN.F.HAK1972a, nine dorsal caudal fin rays are also preserved.

### Postcoelomic Bone

The postcoelomic bone is located between the cloaca and the anal fin, forming the posterior delimitation of the body cavity. It is an elongate, hockey-stick-shaped bone, which is typical of pycnodonts (Fig. 8).

### Cloaca

The cloaca is roofed by two comma-shaped scales (Fig. 8). The precloacal ventral ridge scale has two posterior-pointing spines, with one larger dorsal spine lying dorsal to a smaller ventral spine. The postcloacal ventral ridge scale has four spines, of which the first three are strongly curved and forward pointing and the last, most posterior spine is straight and located in a posteroventral angle pointing caudally. A small, triangular scale roofing the cloaca is all that is preserved in MNHN.F.HAK2001.

### Axial Skeleton

Unfortunately, either the posterior-most portion of the skeleton including the caudal fin is disarticulated (MNHN.F.HAK2003, MNHN.F.HAK2001) or the anterior-most part (MNHN.F.HAK1972a, MNHN.F.HAK1972b) is not preserved, so that it is not possible to establish the exact number of vertebral elements unambiguously. In the holotype, 29 vertebrae are present, and 30 are present in MNHN.F.HAK2001 (juvenile specimen) counting the neural arcocentra and neural spines. In MNHN.F.HAK1972a and MNHN.F.HAK1972b, only 25 vertebrae are preserved. The anterior-most six neural spines in the holotype are autogenous, whereas only four autogenous spines are discernible in MNHN.F.HAK2001. The arcocentra surround the notochord completely in the two adult specimens but only partially in the supposed juvenile specimen (MNHN.F.HAK2001). Each arcocentrum bears a small medial-dorsal extension with large lateral projections with rounded margins that are in very close contact with adjacent arcocentra. The arcocentra closest to the caudal fin are ornamented with multiple striations. The corresponding arcocentra in MNHN.F.HAK2001 are not as closely in contact with each other as seen in the holotype. The arcocentra are tightly connected to each other by zygapophyses with multiple pointed interdigitations reinforcing the vertebral column.

All neural and hemal spines have anterior and posterior sagittal flanges when preserved, which cover the proximal half of the spines. In most specimens, the posterior flanges are very poorly preserved, however, and are only visible in some postabdominal areas in MNHN.F.HAK2001 and MNHN.F.HAK1972. The neural and hemal spines in MNHN.F.HAK1972b are identical to those in the holotype, with the exception that it can be seen that the dorsal tips of the neural spines bend backward and then forward to form a chevron-shaped tip. This bending is most pronounced in the anterior neural spines, and they become increasingly straighter in a caudal direction. A small split in the rock is present going through the middle of this chevron tip, which indicates that this is not a genuine anatomical character, but a taphonomic artifact producing this peculiar shape.

Ribs are difficult to identify. In the holotype, seven pairs of ribs are preserved, whereas only five pairs of ribs, which terminate just dorsal to the ventral ridge scales, are present in MNHN.F.HAK2001.

### Squamation

**Ridge Scales**—The morphology, and number, of ridge scales (contour scales) has high taxonomic and systematic importance for pycnodont fishes (e.g., Nursall, 1996; Poyato-Ariza and Wenz, 2004). In the holotype, 10 dorsal ridge scales are preserved. The number of spines on these scales changes from one to two posteriorly. In MNHN.F.HAK2001, 16 or 17 dorsal ridge scales without spines are present. The first 10 scales are oval, whereas the remaining scales are more elongate and are in very close contact with each other. The best-preserved dorsal ridge scales are the 15 scales preserved in MNHN.F.HAK1972b (Figs. 2, 9). These are elongate, almost bar-like with backward-pointing spines. The number of spines varies from as little as three to as many as six in this specimen. The final dorsal ridge scale just anterior to the dorsal fin is the largest in the dorsal ridge scale series. It has one large, backward-pointing spine protruding from its base. The anterior-most dorsal ridge scale (dorsal ridge scale 1) is incorporated onto the cranium in close contact with the dermosupraoccipital and does not differ morphologically from the subsequent dorsal ridge scales when present (e.g., in the holotype).

It is not possible to establish the exact number of ventral ridge scales in either adult specimen (MNHN.F.HAK2003, MNHN.F.HAK1972). In MNHN.F.HAK2003, 12 ventral ridge scales are preserved, of which 11 are precloacal and one a postcloacal scale. In MNHN.F.HAK2001, 14 ventral ridge scales are preserved, with 12 precloacal and two postcloacal scales. Many of these scales have two backward-curving spines (Fig. 8). The spines have become disarticulated from some scales, so the true number of ventral ridge scales and/or spines is hard to determine. Some spines become much more strongly curved in certain scales, such as the posterior spine in the scale anterior to the cloaca and the two spines located below the cleithrum. Precloacal scales in MNHN.F.HAK2001 are elongate with three to four backward-pointing spines, which are distributed along the ventral margin of the entire scale. The anterior-most postcloacal scale is triangular in form and has two backward-pointing spines preserved at the tip of the scale. A curious sigmoid-shaped bone located between the postcloacal ventral ridge scale and the anal fin is present. This could represent a poorly preserved postcloacal ventral ridge scale.

In MNHN.F.HAK1972a, there seems to be an unusual ossification preserved posterior and ventral to the few skull remains (parietal, orbit, preoperculum). It has a broad, wavy fan-shaped head with a narrow but still comparatively broad base with a convex ventral margin. Due to its position, this could be the ventral view of a ventral ridge scale, something that is very rarely preserved in pycnodonts.

**Flank Scales**—The flank squamation is poorly preserved, and most scale bars are slightly disarticulated or displaced, so the exact number of scale rows cannot be established unambiguously. It is nevertheless evident that no scale rows persisted onto the caudal peduncle but are restricted to the body (complete body scale cover). All scale rows are oriented in the same direction, being arranged obliquely in an anterior direction. Whereas most of the scales are reduced such that only scale bars cover most of the body, forming a lattice pattern with the neural and hemal spines, completely ossified scales occur only abdominally. The scale bars are continuous dorsally and ventrally without any bifurcating pattern. Additionally, the scale bars do not extend between the lepidotrichia as found in more basal pycnodonts. The ornamentation of the abdominal scales is reticulate. This squamation pattern differs from all types introduced by Nursall (1996) and described by Poyato-Ariza and Wenz (2004). However, it does seem to match the pattern seen in *‘Nursallia’ goedeli*, which Kriwet (2001a) describes as striate.

### Sensory Lines

The sensory lines are quite poorly preserved or obscured by displaced bones in all specimens, but there are a few dermal bones where they can be seen clearly. The supraorbital sensory line is quite clearly visible on the ventral margin of the parietal just above the orbit. The preservation of the mesethmoid and preparietal is not sufficient to identify the supraorbital canal unambiguously. Following the supraorbital canal in an anteroposterior direction into the posteroventral corner of the dermopterosphenotic reveals the temporal portion of the sensory line. Another sensory canal extends across the anterodorsal region of the dermopterosphenotic and represents the parietal canal. The parietal canal travels dorsally into the postparietal. The occipital canal cannot be observed in any of the specimens studied here. The infraorbitals are only preserved in MNHN.F.HAK1972b, and the supposed sensory line that seems to be present in the most posterior infraorbital looks to be a case of taphonomic processes rather than a genuine sensory line. As a result, the path of the sensory line through the infraorbitals is unknown.

A sensory line extends down the posterior region of the preoperculum as best seen in MNHN.F.HAK2001, whereas that part of the preoperculum is overlapped by the operculum in the holotype. In both specimens, the preopercular canal continues along the ventral border of the preoperculum and into the quadrate. The mandibular canal cannot be seen in MNHN.F.HAK2001 due to poor preservation, but pores can be seen in the dentalosplenial of the holotype.

## DISCUSSION

### Taxonomic Position of the New Taxon within Pycnodontiformes

Using the characters from the phylogenetic database of Poyato-Ariza and Wenz (2002), we can determine roughly where the new taxon fits within the pycnodont clade. The diamond-shaped body is similar to that of the mesturid *Arduafrons*, and one important structure is preserved, particularly in MNHN.F.HAK2003, which is the presence of a postparietal process, revealing it to be a member of the family Pycnodontidae. The postparietal process is an autapomorphy, but other analogous characters are present that in combination also lend support to it being a pycnodontid. These include the absence of suborbitals, the presence of two incisiform teeth on the dentalosplenial and premaxilla, and the presence of an edentulous reniform maxilla.

### Taxonomic Position of the New Taxon within Pycnodontidae

Unfortunately, very few teeth, particularly those of the vomer, are preserved on any of the specimens, so these characters are not applicable in determining where this specimen fits into the phylogenetic scheme of pycnodontids. The broad, incisiform premaxillary and dentalosplenial teeth are reminiscent of what is found in other pycnodontids, such as Pycnodontinae or various members of the subfamily Nursallinae (its status as a valid subfamily within Pycnodontidae remains, however, ambiguous; Cawley and Kriwet, 2018). A posteriorly exposed endocranium, which is an autapomorphy of the subfamily Pycnodontinae (Poyato-Ariza and Wenz, 2002), unfortunately is not preserved or identifiable in any of the specimens. However, there are additional characters that support the species inclusion within Pycnodontinae, such as the complex interdigitating zygapophyses of adjacent arcocentra, the incomplete ossification of the dorsal scales to scale bars, and 10–14 ventral ridge scales (12 in MNHN.F.HAK2003). The character combination (e.g., extremely elongate fourth to sixth dorsal fin rays, very prognathous snout in adults, unique squamation pattern, very large operculum, two different tooth crown morphologies in the premaxillae and dentalosplenials, etc.) supports its status as a new taxon within Pycnodontiformes and Pycnodontidae.

### Notes on the Caudal Endoskeleton

The specimen assigned to *Flagellipinna rhomboides*, gen. et sp. nov., that is figured on page 93 of Gayet et al. (2012) has a more completely preserved caudal fin and can be used to supplement the description here. Because the specimen belongs to the personal collection of Pierre Abi Saad (Gayet et al., 2012), our description will be based on the photo alone, which is why it is not part of the main description of *Flagellipinna rhomboides*, gen. et sp. nov. The posterior trailing edge of the caudal fin in this specimen is convex, and the fin rays bifurcate at their distal tips. Six hypochordals seem to be present, with the first three hypochordals being slim bones that broaden out to a fan as seen in MNHN.F.HAK1972b and the most posterior hypochordals being more fan-shaped with a narrow base. The number of epichordals is very low, with just two to three present on the specimen.

### Ontogenetic and Ecological Considerations

The evidence that the specimen MNHN.F.HAK2001 represents a juvenile form is supported by several characters shared with the other three specimens (the two described here and the one figured in Gayet et al., 2012:93), especially with MNHN.FHAK2003: ‘humpbacked’ appearance; similar shape of the preoperculum, dermohyomandibular, metapterygoid, and quadrate; the anterior bicuspid tooth in the premaxilla and incisiform teeth in the dentalosplenial; and the deep insertion of the dorsal and anal pterygiophores into the body.

The morphological differences between MNHN.F.HAK2001 and the other specimens include poorly ossified dermal skull roof bones and mesethmoid, a relatively larger orbit, a shorter preorbital distance, a dorsal fin lacking the whip-like extension that is present in both larger specimens, a notochord that is only partially surrounded by the arcocentra, flank scales that do not cover the ventral flank posterior to the postcoelomic bone, and dorsal ridge scales that lack spines. The first dorsal ridge scale is located farther from the skull roof, in contrast to the condition seen in MNHN.F.HAK2003.

We interpret this combination of similar but slightly different characters in MNHN.F.HAK2001 to show it to be a juvenile or possibly subadult of the new taxon. The transitional state found in some characters compared with their condition in the larger, supposedly adult specimens, such as the extent of the area covered by the flank scales, the degree to which the notochord is surrounded by the arcocentra, distance of first dorsal ridge scale from the posterior margin of the skull roof, and the morphology of the longest dorsal fin ray, are strong indications of ontogenetic development in this specimen.

The Late Cretaceous was a time of great morphological (Marramà et al., 2016) and ecological (Poyato-Ariza, 2005; Poyato-Ariza and Martín-Abad, 2013) diversification among pycnodonts. The new taxon presented here has a typical laterally flattened body that is characteristic of most pycnodonts, which suggests that it lived in complex shallow water habitats but not necessarily reefs (Webb, 1984). The whip-like dorsal fin extension is very similar to that seen in butterflyfishes of the genus *Heniochus* and the Moorish idol, *Zanclus cornutus*, which predominantly live on reef slopes (Myers, 1999; Lieske and Myers, 2009; Allen and Erdmann, 2012); because the depositional environment of Haqel indicates it to be a series of small, deep basins situated between rudist patch reefs in the warm, shallow waters of the Tethys Sea (Roger, 1946; Patterson, 1967; Hückel, 1970; Philip et al., 1993), it seems possible that the new taxon could have occupied seaward slopes off the reef edge and was carried into deeper areas of the basin post mortem, and the specimens thus might represent allochthonous elements.

**FIGURE 10.**
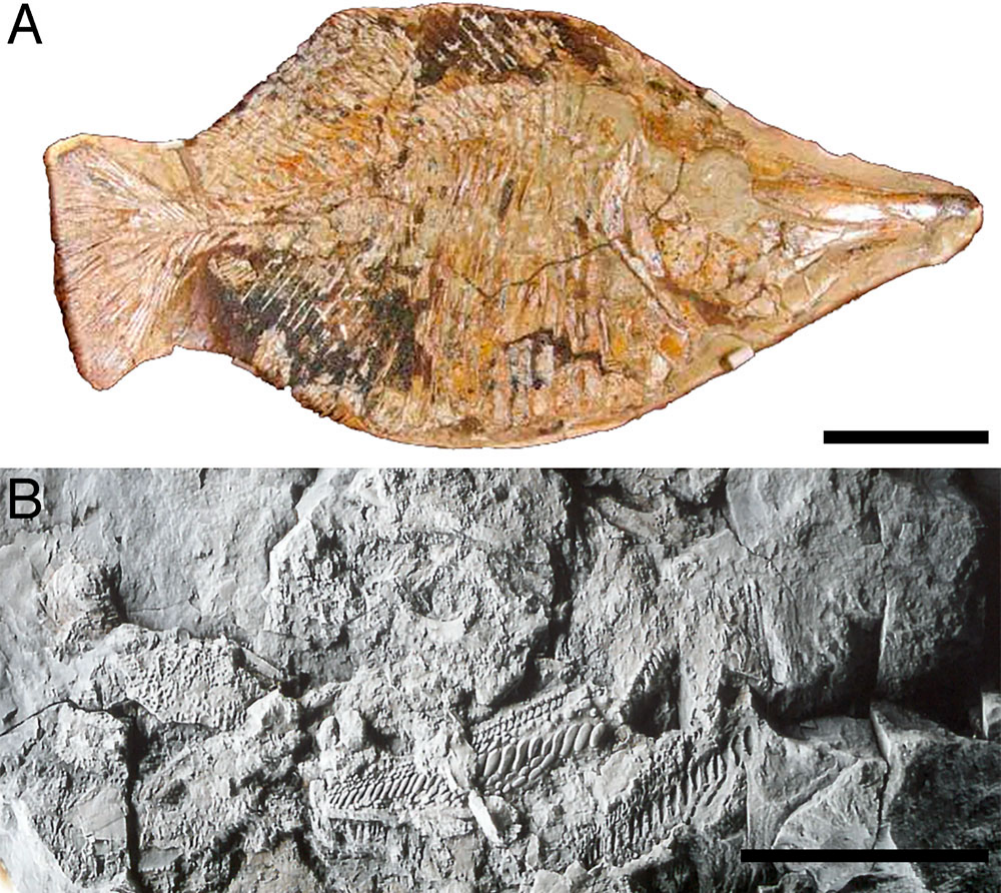
Pycnodont taxa that share prognathism with *Flagellipinna rhomboides*, gen. et sp. nov. **A**, *Iemanja palma*, NSM PV-20384. **B**, *Anomoeodus nursalli*, IPFUB-Uña Pyc 1. Scale bars equal 10 cm (**A**) and 1 cm (**B**).

Whereas the few teeth preserved in the specimens indicate a durophagous diet typical of pycnodonts, the elongate snout seen in MNHN.F.HAK2003 indicates a possible adaptation to crevice feeding as seen in modern actinopterygians such as the butterflyfishes of the genus *Forcipiger*, which feed on polychaetes and other, similar crevice-dwelling invertebrates (Motta, 1988; Ferry-Graham et al., 2001). An even closer modern ecological analog is the labrid *Gomphosus varius*, which uses its elongate snout to feed predominantly on more heavily armored prey that hide in the recesses of reefs (Hiatt and Strasburg, 1960; Randall et al., 1990; Westneat, 2001). The presence of incisiform grasping teeth supports this interpretation. Two other pycnodont taxa may have filled a similar niche, *Iemanja palma* from the Early Cretaceous of Brazil (Poyato-Ariza, 2005) and the genus *Anomoeodus* from the Late Cretaceous of Europe (Kriwet, 2001a), both of which exhibit elongate snouts and may also have fed from crevices of reef habitats (Fig. 10).

However, the Lebanese fossil record for typical crevice-dwelling prey such as bivalves, cirripedes, isopods, echinoderms, and gastropods is spotty at best. Bivalves are quite uncommon, with only four genera known from Haqel: *Exogyra*, *Arca*, *Protocardia*, and a pectinid similar to *Chlamys* (Gayet et al., 2012). Gastropods are extremely rare, with only one genus, *Natica*, present in Haqel (Pictet and Humbert, 1866; Gayet et al., 2012). With the exception of a preserved imprint of tube footprints and tubercles (Gayet et al., 2012:55), echinoids are all but absent from the Lebanese fossil sites.

Such a low number of bivalves, gastropods, and echinoids in the Lebanese Cenomanian fossil beds leads to the conclusion such taxa might have been a low contributor to the marine ecosystem of the Cenomanian of Lebanon. However, the high number of pycnodont species found in the Lebanese fossil sites (Appendix 1), including more traditional durophagous forms such as *Akromystax tilmachiton*, *Nursallia*, and *Cococodus*, and the diversity and widespread distribution of typical pycnodont prey items throughout the fossil record lead one to suggest that such taxa would have also been present in higher numbers and many prey species would have taken to crevice dwelling in order to avoid predation by durophagous fishes. Crevice-dwelling bivalves, gastropods, and echinoids would have been a rich food source for pycnodonts that could reach them.

Due to the transitional state of various morphological characters and overall total length of MNHN.F.HAK2001, we interpret it to be a late juvenile/subadult of the new species. We also assume that it was already durophagous in its diet because it possesses strongly molariform teeth similar to those seen in MNHN.F.HAK2003. The shape of the skull is one of the most intriguing differences between this specimen and MNHN.F.HAK2003. The skull of MNHN.F.HAK2001, with its deep and quite short lower jaw, as well as the relatively short preorbital length, is similar to the cranial morphology seen in many other, adult pycnodonts, suggesting that the juvenile might have been a generalized feeder on shelled prey in complex, structured habitats. This could also suggest that it might have lived in shallower waters than did the adult, and could have moved near the continental slope as it matured. Specimen MNHN.F.HAK2003, on the other hand, suggests a dramatic ontogenetic change in the skull, such as it being obliquely oriented with respect to the body axis and the jaws becoming more gracile. Such a jaw shape is ideal for crevice feeding and suggests that the skull changes in orientation and morphology throughout the ontogeny of this species. Ontogenetic changes in ecological niches during the life cycle of fish are common today in a variety of ways. Ontogenetic shifts in diet are usually accompanied by changes in tooth and jaw morphologies as fishes mature (Stoner and Livingston, 1984; Howes and Sanford, 1987; Wainwright and Richard, 1995; Mullaney and Gale, 1996; Hernandez and Motta, 1997; Piet, 1998; Huskey and Turingan, 2001; Sidnei and Goitein, 2003; Russo et al., 2007).

Perhaps the most relevant example of morphological change during ontogeny is in the crevice-feeding labrid *Gomphosus varius*, in which the snout of the juvenile is significantly shorter than that of the adult and the degree of snout elongation is far less extreme (Myers, 1999), which indicates that crevice feeding is also a specialization that appears later in life. The correlation between ontogenetic processes with ecomorphology is well documented in extant fishes and gives further support to the interpretation that similar processes played out in pycnodonts as well, such as in the new Lebanese taxon described herein.

## CONCLUSIONS

*Flagellipinna rhomboides*, gen. et sp. nov., is a recent addition to the high biodiversity of the exceptional pycnodont fauna of the Cenomanian Lebanese fossil beds (Appendix 1). Anatomical characters such as the presence of a postparietal process indicate that this taxon is a pycnodontid, which are well represented in the Cenomanian of Lebanon (Appendix 1).

The characteristics distinguishing *Flagellipinna*, gen. nov., from other pycnodonts is its acute anterior profile with a diamond-shaped body; a reduced operculum, which is nevertheless broader than typical pycnodont opercula and lies posterior to the dermohyomandibular and the preoperculum; a narrow and elongate cleithrum with two limbs; a posteroventral spine of the postcloacal scale, which is straight and backward pointing alongside three forward-pointing spines; a dorsoventrally flattened skull with a prognathous snout; a deep insertion of the dorsal and anal pterygiophores; and whip-like extended dorsal fin rays reminiscent of modern coral reef fishes such as *Zanclus cornutus*.

A combination of inferences from the specimens, the paleoenvironment of Haqel, and the invertebrate fauna that were found alongside *Flagellipinna*, gen. nov., suggests that it possibly inhabited reef slopes and fed from crevices on elusive armored prey such as bivalves, echinoderms, and gastropods. The interpretation of the smallest specimen of *Flagellipinna*, gen. nov., being a juvenile raises interesting questions regarding ontogeny and change in ecology during growth of this fish. The juvenile has an even deeper body shape than the adult along with a shift in skull position and shape, indicating that it was a more typical durophagous pycnodont that lived in shallower reef habitats and may have moved into deeper waters to feed from the crevices of the reef edge as it became older. Of course, more specimens of *Flagellipinna*, gen. nov., are necessary in order to confirm the validity of these morphological changes throughout its ontogeny, but this interpretation is supported by similar ontogenetic changes observed in modern taxa.

The Lebanese Cenomanian pycnodont fauna contains some of the most diversified assemblages of non-teleost actinopterygians in the Cretaceous. Such a diverse range of forms must have had a wide range of ecological requirements and life histories. *Flagellipinna rhomboides*, gen. et sp. nov., is not only another new species from this extraordinary assemblage, but the specimens described here also hint at how these fishes could have filled a particular niche in the Haqel ichthyofauna and how that niche may have changed during its life span.

## Appendix

**APPENDIX 1.** List of all complete pycnodont taxa found in the Lebanese Cenomanian deposits.

Taxon	Family	Location
*Acrorhinichthys poyatoi* Taverne and Capasso, 2015b	Pycnodontidae	Ein Nammoura, Haqel
*Akromystax tilmachiton* Poyato-Ariza and Wenz, 2005	Pycnodontidae	Haqel
*Coccodus armatus* Pictet, 1850	Coccodontidae	Haqel
*Coccodus insignis* Hay, 1903	Coccodontidae	Hjoûla, Haqel
*Corusichthys megacephalus* Taverne and Capasso, 2014b	Coccodontidae	Haqel
*Ducrotayichthys cornutus* Taverne and Capasso, 2015a	Gladiopycnodontidae	Haqel
*Gebrayelichthys uyenoi* Nursall and Capasso, 2004	Gebrayelichthyidae	Hjoûla, Haqel
*Gebrayelichthys vexillarius* Nursall and Capasso, 2004	Gebrayelichthyidae	Haqel
*Gladiopycnodus byrnei* Marramà et al., 2016	Gladiopycnodontidae	Hjoûla
*Gladiopycnodus karami* Taverne and Capasso, 2013a	Gladiopycnodontidae	Haqel
*Haqelpycnodus picteti* Taverne and Capasso, 2018a	Pycnodontidae	Haqel
*Hayolperichthys pectospinus* Taverne and Capasso, 2015a	Gladiopycnodontidae	Haqel
*Hensodon spinosus* Hennig, 1907	Coccodontidae	Haqel
*Ichthyoceros spinosus* Gayet, 1984	Gladiopycnodontidae	Haqel
*Joinvillichthys kriweti* Taverne and Capasso, 2014a	Gladiopycnodontidae	Hjoûla, Haqel
*Joinvillichthys lindstroemi* Davis, 1890	Gladiopycnodontidae	Hjoûla, Haqel
*Libanopycnodus wenzi* Taverne and Capasso, 2018b	Pycnodontidae	Ein Nammoura
*Maraldichthys verticalis* Taverne and Capasso, 2014c	Gebrayelichthyidae	Haqel
*Monocerichthys scheuchzeri* Taverne and Capasso, 2013a	Gladiopycnodontidae	Ein Nammoura, Hjoûla
‘*Nursalia*’ *goedeli* Heckel, 1854	Pycnodontidae	Haqel
*Nursallia tethysensis* Capasso et al., 2009	Pycnodontidae	Ein Nammoura, Hjoûla
*Palaeobalistum* sp.	Pycnodontidae	Hjoûla, Haqel
*Pankowskichthys libanicus* Taverne and Capasso, 2014a	Gladiopycnodontidae	Ein Nammoura, Haqel
*Paracoccodus woodwardi* Taverne and Capasso, 2014b	Coccodontidae	Haqel
*Proscinetes*, sp. indet. Forey et al., 2003	Pycnodontidae	Ein Nammoura
*Rhinopycnodus gabriellae* Taverne and Capasso, 2013b	Pycnodontidae	Haqel
*Rostropycnodus gayeti* Taverne and Capasso, 2013a	Gladiopycnodontidae	Haqel
*Sigmapycnodus giganteus* Taverne and Capasso, 2018b	Pycnodontidae	Haqel
*Stenoprotome hamata* Hay, 1903	Gladiopycnodontidae	Haqel
*Trewavasia carinata* Davis, 1887	Coccodontidae	Haqel
*Tricerichthys wenzi* Taverne and Capasso, 2015a	Gladiopycnodontidae	Haqel

## References

[CIT0001] Allen G. R., and Erdmann M. V. 2012 Reef Fishes of the East Indies, Volume 1. Tropical Reef Research, Perth, Australia, 1292 pp.

[CIT0002] Arratia G., and Schultze H. P. 1992 Reevaluation of the caudal skeleton of certain actinopterygian fishes: III. Salmonidae. Homologization of caudal skeletal structures. Journal of Morphology 214:187–249. doi: 10.1002/jmor.1052140209 29865606

[CIT0003] Berg L. S. 1937 A classification of fish-like vertebrates. Bulletin de l’Académie des Sciences de l’URSS, Classe des Sciences Mathématiques et Naturelles 4:1277–1280.

[CIT0004] Capasso L. L., Abi Saad P., and Taverne L. 2009 *Nursallia tethysensis* sp. nov., a new pycnodont fish (Neopterygii: †Halecostomi) from the Cenomanian of Lebanon. Bulletin de l’Institut royal des Sciences naturelles de Belgique, Sciences de la Terre 79:117–136.

[CIT0005] Capasso L. L., Taverne L., and Nohra R. 2010 A re-description of *Hensodon spinosus*, a remarkable coccodontid fish (Actinopterygii, †Pycnodontiformes) from the Cenomanian (Late Cretaceous) of Haqel, Lebanon. Bulletin de l’Institut royal des Sciences naturelles de Belgique, Sciences de la Terre 80:145–162.

[CIT0006] Cavin L., Forey P. L., and Lecuyer C. 2007 Correlation between environment and Late Mesozoic ray-finned fish evolution. Palaeogeography, Palaeoclimatology, Palaeoecology 245:353–367. doi: 10.1016/j.palaeo.2006.08.010

[CIT0007] Cawley J. J., and Kriwet J. 2018 A new pycnodont fish, *Scalacurvichthys naishi* gen. et sp. nov., from the Late Cretaceous of Israel. Journal of Systematic Palaeontology 16:659–673. doi: 10.1080/14772019.2017.1330772 29551954 PMC5849399

[CIT0008] Cawley J. J., Marramà G., Carnevale G., and Kriwet J. 2018 A quantitative approach to determine the taxonomic identity and ontogeny of the pycnodontiform fish *Pycnodus* (Actinopterygii, Neopterygii) from the Eocene of Bolca Lagerstätte, Italy. PeerJ 6:e4809. 10.7717/peerj.4809 PMC5961631 29796348

[CIT0009] Cope E. D. 1887 Zittel’s Manual of Palæontology. American Naturalist 21:1014–1019.

[CIT0010] Davis J. W. 1887 The fossil fishes of the Chalk of Mount Lebanon in Syria. Scientific Transactions of the Royal Dublin Society 3:457–636.

[CIT0011] Davis J. W. 1890 On a new species of *Coccodus* (*C. lindstroemi*, Davis). The Quarterly Journal of the Geological Society of London 46:565–568. doi: 10.1144/GSL.JGS.1890.046.01-04.36

[CIT0012] Ferry-Graham L. A., Wainwright P. C., Hulsey C. D., and Bellwood D. R. 2001 Evolution and mechanics of long jaws in butterflyfishes (Family Chaetodontidae). Journal of Morphology 248:120–143. doi: 10.1002/jmor.1024 11304744

[CIT0013] Forey P. L., Yi L., Patterson C., and Davies C. E. 2003 Fossil fishes from the Cenomanian (Upper Cetaceous) of Namoura, Lebanon. Journal of Systematic Palaeontology 1:227–330. doi: 10.1017/S147720190300107X

[CIT0014] Gale A. S. 2000 The Cretaceous world; in pp. 1–19 in Culver S. J. and Rawson P. F. (eds.), Biotic Response to Global Change: The Last 145 Million Years. Cambridge University Press, Cambridge, U.K.

[CIT0015] Gayet M. 1984 *Ichthyoceros spinosus* nov. gen., nov. sp., du Cénomanien inférieur de Hakel (Liban) et ses affinités avec le genre *Trewavasia* (Pisces, Pycnodontiformes, Coccodontidae). Bulletin du Muséum national d’Histoire naturelle 3:287–307.

[CIT0016] Gayet M., Abi Saad P., and Gaudant O. 2012 Les fossiles du Liban: Memoire du Temps. Éditions Desiris, Meolan-Revel, France, 184 pp.

[CIT0017] Haq B. U., Hardenbol J., and Vail P. 1987 Chronology of ﬂuctuating sea levels since the Triassic. Science 235:1156–1167. doi: 10.1126/science.235.4793.1156 17818978

[CIT0018] Hay O. P. 1903 On a collection of Upper Cretaceous fishes from Mount Lebanon, Syria, with descriptions of four new genera and nineteen new species. Bulletin of the American Museum of Natural History 19:395–452.

[CIT0019] Heckel J. 1854 Über den Bau und die Eintheilung der Pycnodonten, nebst kurzer Beschreibung einiger neuen Arten derselben. Sitzungsberichte der kaiserlichen Akademie der Wissenschaften, Mathematisch-Naturwissenschaftliche Klasse 12:433–464.

[CIT0020] Hennig E. 1907 Ueber einige Pyknodonten vom Libanon. Centralblatt für Mineralogie, Geologie und Palâontologie 1907:360–371.

[CIT0021] Hernandez L. P., and Motta P. J. 1997 Trophic consequences of differential performance: ontogeny of oral jaw-crushing performance in the sheepshead, *Archosargus probatocephalus* (Teleostei, Sparidae). Journal of Zoology 243:737–756. doi: 10.1111/j.1469-7998.1997.tb01973.x

[CIT0022] Hiatt R. W., and Strasburg D. W. 1960 Ecological relationships of the fish fauna on coral reefs of the Marshall Islands. Ecological Monographs 30:65–127. doi: 10.2307/1942181

[CIT0023] Howes G. J., and Sanford C. P. J. 1987 Oral ontogeny of the Ayu, *Plecoglossus altivelis* and comparisons with the jaws of other salmoniform fishes. Zoological Journal of the Linnean Society 89:133–169. doi: 10.1111/j.1096-3642.1987.tb00653.x

[CIT0024] Hückel U. 1970 Die Fishschiefer von Haqel and Hadjoula in der Oberkreide des Libanon. Neues Jahrbuch für Geologie und Paläontologie, Abhandlungen 135:113–149.

[CIT0025] Huskey S. H., and Turingan R. G. 2001 Variation in prey-resource utilization and oral jaw gape between two population of largemouth bass, *Micropterus salmoides*. Environmental Biology of Fishes 61:185–194. doi: 10.1023/A:1011095526939

[CIT0026] Huxley T. H. 1880 On the application of the laws of evolution to the arrangement of the Vertebrata, and more particularly of the Mammalia. Proceedings of the Scientific Meetings of the Zoological Society of London 1880:649–661.

[CIT0027] Jollie M. 1962 Chordate Morphology. Reinhold, New York, 504 .

[CIT0028] Kriwet J., 2001a A comprehensive study of pycnodont fishes (Neopterygii, Pycnodontiformes): morphology, taxonomy, functional morphology, phylogeny, and palaeobiogeography. Ph.D. dissertation, Humboldt University, Berlin, Germany, 582 pp.

[CIT0030] Kriwet J., and Schmitz L. 2005 New insight into the distribution and palaeobiology of the pycnodont fish *Gyrodus*. Acta Palaeontologica Polonica 50:49–56.

[CIT0031] Lieske E., and Myers R. 2009 Coral Reef Fishes. Princeton University Press, Princeton, New Jersey, 400 .

[CIT0032] Longbottom A. E. 1984 New Tertiary pycnodonts from the Tilemsi valley, Republic of Mali. Bulletin of the British Museum (Natural History) 38:1–26.

[CIT0033] Marramà G., Villier B., Dalla Vecchia F. M., and Carnevale G. 2016 A new species of *Gladiopycnodus* (Coccodontoidea, Pycnodontomorpha) from the Cretaceous of Lebanon provides new insights about the morphological diversification of pycnodont fishes through time. Cretaceous Research 61:34–43. doi: 10.1016/j.cretres.2015.12.022

[CIT0034] Motta P. J. 1988 Functional morphology of the feeding apparatus of ten species of Pacific butterflyfishes (Perciformes, Chaetodontidae): an ecomorphological approach. Enviromental Biology of Fishes 22:39–67. doi: 10.1007/BF00000543

[CIT0035] Mullaney M. D. Jr., and Gale L. D. 1996 Ecomorphological relationships in ontogeny: anatomy and diet in gag, *Mycteroperca microlepis* (Pisces: Serranidae). Copeia 1996:167–180. doi: 10.2307/1446952

[CIT0036] Myers R. F. 1999 Micronesian Reef Fishes: A Comprehensive Guide to the Coral Reef Fishes of Micronesia, third revised and expanded edition. Coral Graphics, Barrigada, Guam, 330 pp.

[CIT0037] Nursall J. R. 1996 The phylogeny of pycnodont fishes; pp. 125–152 in Arratia G. and Viohl G. (eds.), Mesozoic Fishes: Systematics and Paleoecology. Dr. F. Pfeil, Munich, Germany.

[CIT0038] Nursall J. R., and Capasso L. 2004 *Gebrayelichthys* (novum), an extraordinary genus of neopterygian fishes from the Cenomanian of Lebanon; pp 317–340 in Arratia G. and Tintori A. (eds.), Mesozoic Fishes 3: Systematics, Paleoenvironments and Biodiversity. Dr. F. Pfeil, Munich, Germany.

[CIT0039] Patterson C. 1967 New Cretaceous berycoid fishes from the Lebanon. Bulletin of the British Museum (Natural History) 14:69–109.

[CIT0041] Philip J., Babinot J. F., Tronchetti G., Fourcade E., Ricou L. E., Guiaud R., Bellion Y., Herbin J. P., Combes P. E., Conee J. J., and Dercourt J. 1993 Late Cenomanian palaeoenvironments (94–92 Ma); pp. 153–178 in Dercourt J., Ricou L. E., and Vrielynck B. (eds.), Atlas Tethys Palaeoenvironmental Maps. Gauthier-Villars, Paris.

[CIT0042] Pictet F. J. 1850 Description de quelques poissons fossiles du Mont Liban. Imprimerie De Jules-Guillaume Fick, Geneva, 59 pp.

[CIT0043] Pictet F. J., and Humbert A. 1866 Nouvelles recherches sur les poissons fossiles du Mont Liban. George, Geneva, 114 .

[CIT0044] Piet G. J. 1998 Ecomorphology of a size-structured tropical freshwater fish community. Environmental Biology of Fishes 51:67–86. doi: 10.1023/A:1007338532482

[CIT0045] Poyato-Ariza F. J. 2005 Pycnodont fishes: morphologic variation, ecomorphologic plasticity, and a new interpretation of their evolutionary history. Bulletin of the Kitakyushu Museum of Natural History and Human History, series A (Natural History) 3:169–184.

[CIT0046] Poyato-Ariza F. J., and Martín-Abad H. 2013 History of two lineages: comparative analysis of the fossil record in Amiiformes and Pycnodontiformes (Osteichthyes, Actinopterygii). Spanish Journal of Palaeontology 28:79–90.

[CIT0047] Poyato-Ariza F. J., and Wenz S. 2002 A new insight into pycnodontiform fishes. Geodiversitas 24:139–248.

[CIT0048] Poyato-Ariza F. J., and Wenz S. 2004 The new pycnodontid fish genus *Turbomesodon*, and a revision of *Macromesodon* based on new material from the Lower Cretaceous of Las Hoyas, Cuenca, Spain; pp. 341–378 in Arratia G. and Tintori A. (eds.), Mesozoic Fishes 3: Systematics, Paleoenvironments and Biodiversity. Dr. F. Pfeil, Munich, Germany.

[CIT0049] Poyato-Ariza F. J., and Wenz S. 2005 *Akromystax tilmachiton* gen. et sp. nov., a new pycnodontid fish from the Lebanese Late Cretaceous of Haqel and En Nammoura. Journal of Vertebrate Paleontology 25:27–45. doi: 10.1671/0272-4634(2005)025[0027:ATGESN]2.0.CO;2

[CIT0050] Poyato-Ariza F. J., Talbot M. R., Fregenal-Martínez M. A., Melendez N., and Wenz S., 1998 First isotopic and multidisciplinary evidence for nonmarine coelacanths and pycnodontiform fishes: palaeoenvironmental implications. Palaeogeography, Palaeoclimatology, Palaeoecology 144:65–84. doi: 10.1016/S0031-0182(98)00085-6

[CIT0051] Randall J. E., Allen G. R., and Steene R. C. 1990 Fishes of the Great Barrier Reef and Coral Sea. University of Hawaii Press, Honolulu, Hawaii, 557 pp.

[CIT0052] Regan C. T. 1923 The skeleton of *Lepidosteus*, with remarks on the origin and evolution of the lower neopterygian fishes. Proceedings of the Zoological Society of London 1923:445–461.

[CIT0053] Roger J. 1946 Les invertébrés des couches à poissons du Crétacé supérieur du Liban. Étude paléobiologique des gisements. Mémoires de la Société géologique de France (Nouvelle Série) 51:1–92.

[CIT0054] Russo T., Costa C., and Cataudella S. 2007 Correspondence between shape and feeding habit changes throughout ontogeny of gilthead sea bream *Sparus aurata* L., 1758. Journal of Fish Biology 71:629–656. doi: 10.1111/j.1095-8649.2007.01528.x

[CIT0055] Schultze H. P. 1993 The head skeleton of fishes; pp. 189–254 in Hanken J. and Hall B. K. (eds.), The Skull, Volume 2. The University of Chicago Press, Chicago, Illinois.

[CIT0056] Schultze H. P., and Arratia G. 1986 Reevaluation of the caudal skeleton of actinopterygian fishes: I. *Lepisosteus* and *Amia*. Journal of Morphology 190:215–241. doi: 10.1002/jmor.1051900206 29945362

[CIT0057] Schultze H. P., and Arsenault M. 1985 The panderichthyid fish *Elpistostege*: a close relative of tetrapods. Palaeontology 28:293–309.

[CIT0058] Seton M., Gaina C., Müller R. D., and Heine C. 2009 Mid-Cretaceous seaﬂoor spreading pulse: fact or ﬁction? Geology 37:687–690. doi: 10.1130/G25624A.1

[CIT0059] Sidnei E. L.-J., and Goitein R. 2003 Ontogenetic diet shifts of a Neotropical catfish, *Pimelodus maculatus* (Siluriformes, Pimelodidae): an ecomorphological approach. Environmental Biology of Fishes 68:73–79. doi: 10.1023/A:1026079011647

[CIT0060] Stoner A. W., and Livingston R. J. 1984 Ontogenetic patterns in diet and feeding morphology in sympatric sparid fishes from sea-grass meadows. Copeia 1984:174–187. doi: 10.2307/1445050

[CIT0061] Taverne L., and Capasso L. 2013a Gladiopycnodontidae, a new family of pycnodontiform fishes from the Late Cretaceous of Lebanon, with the description of three genera. European Journal of Taxonomy 57:1–30.

[CIT0062] Taverne L., and Capasso L. 2013b Osteology and relationships of *Rhinopycnodus gabriellae* gen. et sp. nov. (Pycnodontiformes) from the marine Late Cretaceous of Lebanon. European Journal of Taxonomy 67:1–14.

[CIT0063] Taverne L., and Capasso L. 2014a On the ‘*Coccodus*’ *lindstroemi* species complex (Pycnodontiformes, Gladiopycnodontidae) from the marine Late Cretaceous of Lebanon, with the description of two new genera. European Journal of Taxonomy 101:1–27.

[CIT0064] Taverne L., and Capasso L. 2014b Osteologie et phylogenie des Coccodontidae, une famille remarquable de poissons Pycnodontiformes du Cretace superieur marin du Liban, avec la description de deux nouveaux genres. Palaeontos 25:3–43.

[CIT0065] Taverne L., and Capasso L. 2014c Osteologie et relations phylogenetiques des Gebrayelichthyidae (Halecostomi, Pycnodontomorpha), une extraordinaire famille de poissons du Cretacee superieur marin du Liban, avec la description d’unnouveau genre. Palaeontos 25:44–68.

[CIT0066] Taverne L., and Capasso L. 2015a New data on the osteology and phylogeny of Gladiopycnodontidae (Pycnodontiformes), a tropical fossil fish family from the marine Upper Cretaceous of Lebanon, with the description of four genera. Geo-Eco-Trop 39:217–246.

[CIT0067] Taverne L., and Capasso L. 2015b Osteology and relationships of *Acrorhinichthys poyatoi* gen. et sp. nov. (Pycnodontiformes) from the marine Late Cretaceous of Lebanon. European Journal of Taxonomy 116:1–30.

[CIT0068] Taverne L., and Capasso L. 2018a Osteology and phylogenetic relationships of *Haqelpycnodus picteti* gen. and sp. nov., a new pycnodont fish genus (Pycnodontidae) from the marine Late Cretaceous tropical sea of Lebanon. Geo-Eco-Trop 42:117–132.

[CIT0069] Taverne L., and Capasso L. 2018b Osteology and relationships of *Libanopycnodus wenzi* gen. et sp. nov. and *Sigmapycnodus giganteus* gen. et sp. nov. (Pycnodontiformes) from the Late Cretaceous of Lebanon. European Journal of Taxonomy 420:1–29.

[CIT0070] Tintori A. 1981 Two new pycnodonts (Pisces, Actinopterygii) from the Upper Triassic of Lombardy (N. Italy). Rivista Italiana di Paleontologia e Stratigrafia 86:795–824.

[CIT0071] Wainwright P. C., and Richard B. A. 1995 Predicting pattern of prey use from morphology of fishes. Environmental Biology of Fishes 44:97–113. doi: 10.1007/BF00005909

[CIT0072] Webb P. W. 1984 Form and function in fish swimming. Scientific American 251:72–83. doi: 10.1038/scientificamerican0784-72

[CIT0073] Westneat M. W. 2001 Labridae. Wrasses, hogfishes, razorfishes, corises, tuskfishes; pp. 3381–3467 in Carpenter K. E. and Niem V. (eds.), The Living Marine Resources of the Western Central Pacific, Bony Fishes Part 4 (Labridae to Latimeridae), Volume 6. Food and Agriculture Organization of the United Nations (FAO), Rome, Italy, 844 pp.

